# A Review of In Situ Methods—Solid Phase Adsorption Toxin Tracking (SPATT) and Polar Organic Chemical Integrative Sampler (POCIS) for the Collection and Concentration of Marine Biotoxins and Pharmaceuticals in Environmental Waters

**DOI:** 10.3390/molecules27227898

**Published:** 2022-11-15

**Authors:** Naghmeh Kamali, Feras Abbas, Mary Lehane, Michael Griew, Ambrose Furey

**Affiliations:** 1 Mass Spectrometry Group, Department Physical Sciences, Munster Technological University (MTU), Rossa Avenue, Bishopstown, T12 P928 Cork, Ireland; 2 HALPIN Centre for Research & Innovation, National Maritime College of Ireland (NMCI), Munster Technological University (MTU), P43 XV65 Ringaskiddy, Ireland; 3 CREATE (Centre for Research in Advanced Therapeutic Engineering) and BioExplore, Munster Technological University (MTU), Rossa Avenue, Bishopstown, T12 P928 Cork, Ireland

**Keywords:** Solid Phase Adsorption Toxin Tracking (SPATT), Polar Organic Chemical Integrative Sampler (POCIS), lipophilic/hydrophilic biotoxin, pharmaceuticals, synthetic sorbent

## Abstract

Solid Phase Adsorption Toxin Tracking (SPATT) and Polar Organic Chemical Integrative Sampler (POCIS) are in situ methods that have been applied to pre-concentrate a range of marine toxins, pesticides and pharmaceutical compounds that occur at low levels in marine and environmental waters. Recent research has identified the widespread distribution of biotoxins and pharmaceuticals in environmental waters (marine, brackish and freshwater) highlighting the need for the development of effective techniques to generate accurate quantitative water system profiles. In this manuscript, we reviewed in situ methods known as Solid Phase Adsorption Toxin Tracking (SPATT) and Polar Organic Chemical Integrative Sampler (POCIS) for the collection and concentration of marine biotoxins, freshwater cyanotoxins and pharmaceuticals in environmental waters since the 1980s to present. Twelve different adsorption substrates in SPATT and 18 different sorbents in POCIS were reviewed for their ability to absorb a range of lipophilic and hydrophilic marine biotoxins, pharmaceuticals, pesticides, antibiotics and microcystins in marine water, freshwater and wastewater. This review suggests the gaps in reported studies, outlines future research possibilities and guides researchers who wish to work on water contaminates using Solid Phase Adsorption Toxin Tracking (SPATT) and Polar Organic Chemical Integrative Sampler (POCIS) technologies.

## 1. Introduction

Determining ‘Quality of the Environment’ is a high priority global challenge. Consistent monitoring and surveillance programmes are required to highlight harmful environmental trends, to detect consequences of pollution on human life, and to initiate remedial action when regulatory limits are exceeded. Water-borne pollutants and biotoxins are key anthropogenic and natural environmental agents which must be reliably, accurately and rapidly detected in both the marine and freshwater ecosystems [1].

Marine biotoxins are naturally occurring metabolites of planktonic and microalgae that enter the food chain so that when ingested by marine organisms and accumulated in their tissues, they pose dangers to humans who consume contaminated seafood [2]. Various toxicological symptoms result from their consumption including gastrointestinal illness such as diarrhoea, nausea or vomiting or neurological effects causing in some cases unconsciousness and death [3]. The aquaculture industry, which is responsible for delivering environmentally sustainable produce devoid of anthropogenic or natural pollutants, maintains consumer safety by the routine monitoring and examination of water and aquaculture products [4]. For example, to counter the worldwide problem of shellfish contamination, produce is subject to biotoxin concentration limits based on strict internationally agreed permissible levels of biotoxins in edible shellfish [5] and consumers are protected by routine monitoring (on a weekly basis) during harvesting periods [6].

Passive sampling is an environmental monitoring technique developed for measuring organic and inorganic compounds in different sample matrices [7]. The concept has proven useful for monitoring pollutants in air, soil and water with applications dating back to 1873 when the technique was first used to estimate atmospheric ozone. In 1927 passive sampling was applied to determine carbon monoxide levels in the atmosphere [8] and in 1973 it was used for the quantitative analysis of the molecular diffusion of gaseous pollutants in the atmosphere [9]. In the 1970s passive sampling was extended to study aqueous environments such as water monitoring system for organic contaminants [1] and to determine pesticides that accumulate in soil and traverse into freshwater environments [10].

Periodic spot sampling regimes cannot quantify average exposure of aquatic organisms to contaminants, nor reveal long-term background level fluctuations and pre-treatment of sample material is necessary to isolate bioavailable fractions of contaminants prior to analysis [11]. Whereas time-integrative continuous in situ sampling, which arose from advances in material science (and in particular molecular imprinting technology) can be used to selectively accumulate and concentrate contaminants over periods of days or weeks. This technique not only serves to pre-concentrate low concentrations of analyte, but also provides a means to observe pollution impacts by mimicking bioaccumulation [11].

In recent decades passive sampling techniques and devices have been developed to monitor organic and inorganic contaminants in aquatic environments. Those technologies have been incorporated into European Commission guidance on surface water chemical monitoring. The aim of the NORMAN network [12] is to investigate and promote passive sampling to complement data gathered by spot sampling, this deployment indicates a future role for passive sampling in the regulatory domain [13].

**Figure 1:**
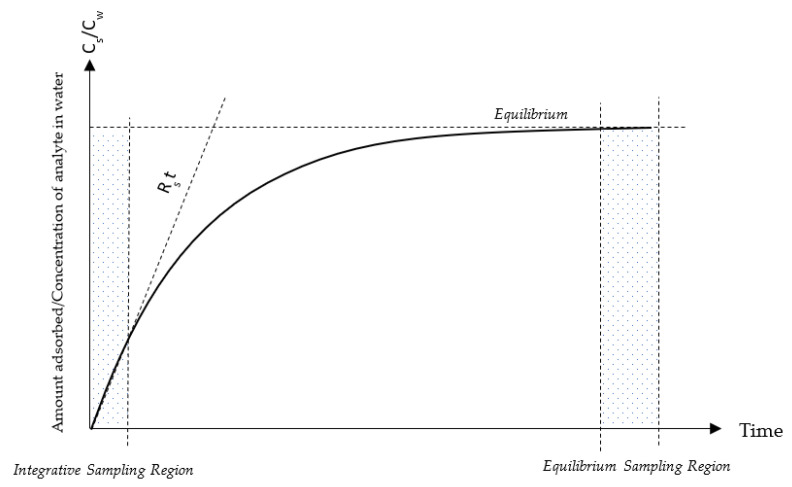
General form of analyte uptake in passive samplers [11].

Passive sampling receiving systems comprise either membrane materials, sorbent phases that act alone, or sorbents that act in combination with partition materials, that accumulate and concentrate contaminant species when exposed to analyte-laden matrices over extended periods. Analyte up-take in samplers is subject to chemical behaviour of the receiving system, characterised by the balance between analyte adsorption and desorption. The general form of this relationship fits Equation (1) [14] shown in Figure 1 where *k*_1_ is the uptake constant, *k*_2_ the elimination constant, *C_s_* analyte concentration in the sampler and *C_w_* analyte compound concentration in water, which is assumed to remain constant. During the integrative sampling phase indicated in Figure 1, when an approximately linear relationship between concentrations of analyte in the water and sorbent exists, thermodynamics are modelled by Equation (2), a simplification of Equation (1).

(1) $$C_{s}=C_{w}\frac{k_{1}}{k_{2}}\left[1-exp\left(-k_{2}\times t\right)\right]$$

(2) $$C_{s}=\frac{R_{s}\times t\times C_{w}}{m_{s}}$$

When first exposed, negligible desorption gives way to a linear rise of analyte concentration. Integrative sampling periods last between several days to weeks and give rise to time-weighted average values for water concentration. Polar Organic Chemical Integrative Sampler (POCIS) and other commercially available passive sampling systems (those commonly applied for freshwater and wastewater quality monitoring), typically operate in this thermodynamic regime. As time passes and available adsorption sites fill, desorption offsets adsorption until the analyte concentration in the receiving material achieves thermodynamic equilibrium, affinity of receiver material for compounds of interest dictate maximum value of *C_s_* achieved. Solid Phase Adsorption Toxin Tracking (SPATT) and generally qualitative sampling systems operate via this regime (although *C_w_* is estimable provided the sampler-water partition coefficient is known) [11]. Regardless of the thermodynamic mode, accumulation within receiving systems selectively concentrates compounds of interest compared to the environmental matrix, thus simplifying pre-treatment of samples [15]. The pre-concentration capacity of passive sampler devices also makes them highly practical for obtaining ultra-trace quantities of chemicals [16]. Performance reference compounds (PRCs) can improve accuracy of quantitative sampling attempts. If the analyte accumulation rate (*R_s_*) for each pollutant captured by the sampler is known, then the time-weighted average concentrations of these compounds in environmental water can be estimated. However, *R_s_* values measured in laboratory conditions differ from those encountered in the field due to local environmental factors (pH, water flow rate, and temperature) therefore *C_w_* estimates will be uncertain. By exposing sorbents to known concentrations of PRCs (compounds not present in the field environment for which sorbent characteristics are known, whose *R_s_* values are affected by environmental factors identical to compounds-of-interest sample rates, and whose desorption follows first-order kinetics analogous to uptake) prior to deployment, the subsequent decay of these PRC levels during deployment indicate in-field *R_s_* values for target analytes.

Proprietary passive samplers most used for water-borne hydrophobic (nonpolar) compounds are Semi-Permeable Membrane Devices (SPMDs), silicone rubber strips and sheets, and low-density polyethylene (LDPE) membranes but ‘Membrane Enclosed Sorptive Sampler’ (MEMSO) devices, non-polar disks for the ‘Chemcatcher’ sampler, and naked chromatography disks (e.g., C_18_, C_8_) may also be used. For hydrophilic (polar) compounds, including biotoxins, POCIS and Chemcatcher disks products are available and suitable naked chromatography disks or ion-exchange resins may also be used. Diffusive gel discs and sheets can be used to sample metals and cations, oxyanions and polar organic compounds [17] however, evidence of extant research [18] indicates that the application of this technology to study marine toxins remains in its infancy.

## 2. Marine Biotoxins

Absorbent substrates employed in passive sampling tools are matched with classes of biotoxins (Table 1 and Table 2) for which they show affinity. These can be categorised according to lipophilic (lipid soluble contaminants) properties, hydrophilic (water soluble contaminants) [19] properties, or for affinity with compounds falling in the category of novel emerging aquatic biotoxins [3].

### 2.1. Lipophilic Biotoxins

Lipophilic biotoxins are naturally occurring compounds that can be found individually or grouped as biotoxin mixtures and are categorised into four groups: (i) okadaic acid (OA) and derivative dinophysistoxins (DTXs), (ii) pectenotoxins (PTXs), (iii) yessotoxins (YTXs) and (iv) azaspiracids (AZAs), based on chemical structure and bioavailability [2] (Table 2 and Supplementary Figure S1). Consumption of lipophilic toxins predominantly causes gastrointestinal symptoms (vomiting, nausea and diarrhoea) [20].

OA, DTXs and PTXs (Supplementary Figure S1) are classified as diarrhetic shellfish poisoning (DSP) biotoxins and were initially detected in the Netherlands in the 1960s through a spate of gastrointestinal incidents caused by the consumption of contaminated mussels [21]. DSPs are heat resistant so remain stable after cooking in some species of shellfish. This group of biotoxins are produced by dinoflagellates (single-celled organisms possessing two flagella, a group that includes the species *Dinophysis* spp. [22]) that occur in large numbers in communities of marine and fresh water plankton [23]. Many dinoflagellates are known to be photosynthetic, but a large fraction of these are in fact mixotrophic, combining photosynthesis with ingestion of prey (phagotrophy [feeding by engulfing a food cell or particle and ingesting it in a phagocytic vacuole] and myzocytosis [“cellular vampirism” as the predatory cell pierces the cell wall and/or cell membrane of the prey cell with a feeding tube, the conoid, and sucks out the cellular content and digests it]) [23,24]. The population of *Dinophysis* spp. can proliferate quickly and spread throughout bays through strong winds and currents [21]. A variety of nutritional and physical parameters can affect the reproduction and survival of microalgae. Nitrogen and phosphate are two essential nutrients: nitrogen plays an important role in the synthesis of protein and chlorophyll; phosphorus is the main constituent in membrane lipids, adenosine triphosphates (ATPs) and DNA [25]. Limitation of nitrogen and phosphorous concentrations would affect the amount of DSP toxin production [26]. Other than nutrition, physical parameters such as salinity, turbidity, temperature and light also affect algae bloom growth and DSP toxin production [26]. The limit value of DSP toxin in bivalves determined by the European Commission is 0.16 µg OA equivalent per kg in test shellfish tissue (Table 1) [27,28]. (The amount of toxins expressed as the amount of okadaic acid that gives the same toxic response followed intraperitoneal administration to mice. This applies similarly for the group of yessotoxins and azaspiracids, respectively [27]).

Okadaic acid (OA), a polycyclic ether, is commonly found in shellfish across Europe, Asia and South America from Spring to late Autumn [29]. Okadaic acid as a lipophilic polyether toxin (Supplementary Figure S1a) that accumulates in the hepatopancreas of shellfish causing illness (e.g., nausea, vomiting, diarrhoea) to consumers of the contaminated shellfish [30,31]. Four metabolites, 19-epi-okadaic acid, belizeanic acid, 11-oxo okadaic acid, 11S-hydroxy and 11R-hydroxy okadaic acid are reported for okadaic acid [32]. These metabolites are produced by the human recombinant cytochrome CYP3A4. These metabolites are similar to okadaic acid and are protein phosphatase PP2A inhibitors [33]. In addition, OA has been studied for the last few decades not only as a diarrhetic toxin [29] but also as a DNA and cellular disruptor that can affect the immune and nervous systems [34]. Furthermore, OA has been studied as a potential carcinogenic agent [35]. OA is an inhibitor of serine/threonine protein phosphatase PP1 and PP2A that increases phosphorylation of some proteins such as myosin. It also affects sodium secretion of intestinal cells resulting in gastro-intestinal fluid accumulation and gastric cramp [31]. OA also reduces activity of PP2A that affects the brain of patients suffering with Alzheimer’s disease [36]. In summary, OA has been shown to be a tumour promoter, genotoxic [31] and hepatotoxic [36].

Liver cells are one of the major targets of OA, causing an interruption to intercellular communication, and precipitating apoptosis in hepatocytes, OA can change the cell morphology of human liver tumour cells and can change the cell proliferation [37]. In addition, to the damage to human cells, OA can induce cytotoxicity and cell death in cervical cancer cell lines [38], affect human kidney cell lines by increasing oxidative DNA damage [39], morphologically change human osteosarcoma cells and cause DNA fragmentation [40]. Availability of two major target of OA, PP1 and PP2A, mixed with OA structures, could be a new design of drug targeting cell. This complex can target the cells by protein phosphatase inhibition [41].

Studies of animal cell lines have reported negative effects on monkey kidney epithelial cells [30]; it decreased l viability of Chinese hamster cells [42] and increased death in Chinese hamster ovary cells [43]. In addition, in vivo studies of okadaic acid toxicology, such as direct injection of OA (≥75 ng/larva) into larva (*Galleria mellonella*) resulting in reduced larval survival (>65%) [44]; studies which show OA promoting tumour growth in Hawaiian green turtle’s fibropapillomatosis [45], and studies demonstrating diarrhetic effects in mouse small intestine via inhibition of the serine/threonine protein phosphatases [46], provide further evidence of OA toxicity.

DTXs, (such as DTX1, DTX2 and their derivative DTX3) (Supplementary Figure S1p–r), belong to the OA structural class of toxins [47] and are known as the diarrhetic lipophilic marine toxins, that during blooms of *Dinophysis* dinoflagellates can be accumulated in shellfish. The 7-O-acyl fatty acid ester of OA in combination with DTX1 and DTX2 are collectively designed as DTX3 [29]. DTX1 acts as protein phosphate inhibitor (PP2A) that acts similarly to OA; DTX2 has reduced protein inhibitor activity [48]. DTX2 typically is known as the late summer toxin occurring during blooms of *Dinophysis acuta* [49].

#### 2.1.1. Yessotoxins (YTXs)

Yessotoxins (YTXs), are a group of lipophilic toxins having 11 contiguous ether rings and one saturated side chain (Supplementary Figure S1b), that show chemical and structural similarities to brevotoxins and ciguatoxins [50]. YTX (Table 2) was first detected in the digestive gland of a bivalve scalene called *Patinopecten yessoensis* (Japanese scallop), in 1986 in Japan [51]. However, more recently YTX was identified in Spain, Italy, Norway, Chile, Norway and New Zealand [52]. YTXs mainly accumulate in the digestive glands of bivalves and can also accumulate in hepatopancreas and muscle tissue [53]. Some YTXs are produced by dinoflagellates directly or are the result of shellfish metabolism, for example YTX and homoYTX, which are produced by dinoflagellates, whilst 45-OH-YTX separates from shellfish as a shellfish metabolite [52].

Yessotoxins (YTX), homoYTX, 45 OH-YTX and 45 OH-homoYTX, are polyether toxins constructed by *Protoceratium reticulatum, Lingulodinium polyedrum* and *Gonyaulax spinifera* [54]. The variety of the toxin profile depends on the origin of the strain. The vast majority of toxin detected in *Protoceratium reticulatum* is YTX. In 2006 *Gonyaulax spinifera* was also verified as an YTX producer [55]. Homo YTX is reported to be produced by *Lingulodinium polyedrum* [56] and has also been detected in a strain of *P. reticulatum* in Spain [54], China [57] and Japan [58].

YTX, is a ladder-shaped polycyclic polyether compound containing two sulphur groups with a high polarity end that allows it to interact with plasma membrane components [50] (Supplementary Figure S1b). The main target of YTXs are scallops and mussels (filter feeders) species which can siphon water in from one side and expel water from the other side while extracting oxygen and foods in between, this filter feeding mechanism in mussels and scallops enables them to accumulate high amounts of YTXs (4.5–65 pg/cell). The concentration of Homo-YTX in this study reported between zero to 0.80 pg/cell in *Lingulodinium polyedrum* and 33.4 pg/cell in *Gonyaulax spinifera* [56].

The individual injection of 100 µg/kg YTX and homoYTX are reported as a lethal concentration in mice [59]. In addition, the neuronal brain damage, liver and pancreas failure and cardiovascular toxicity of YTXs indicate the possible health impact of YTXs on human health [60,61]. The mouse bioassay test shows YTXs produce toxicological symptoms similar to diarrheic shellfish poisoning (DSP) [62]. However, coexisting YTXs and OA changes the toxicological functionality as protein phosphatases 2A (PP2A) inhibits or causing diarrhoea, therefore it hindered YTX to be categorised in the DSP toxin group, although YTX still remains listed as a DSP toxin by accumulating in shellfish predominantly [62,63]. YTX have been recognized as a potent cytotoxins, affecting the neural networks, [64] neuronal death in rats was found following exposure to ≥25 nM YTX for around 48 h [60]. Therefore, the authorized level of yessotoxins has increased to 3.75 mg equivalent per kg shellfish meat (i.e., this is the YTX concentration limit for food as recommended by the European Commission (Table 1) [65].

#### 2.1.2. Azaspiracids (AZAs)

Azaspiracid (Supplementary Figure S1c) was first discovered after the ingestion of contaminated mussels originally from Ireland in 1995 caused human illness (Table 2). Extensive chemical analysis revealed the culprit was a new class of toxin [66,67]. Since then, more than 60 analogues of AZA have been reported [68,69,70] and almost 21 different type of AZA characterized [71]. AZA1 was first reported in 1998 in blue mussels, AZA2 with an extra methyl group on C8, AZA3 with a less methyl group in C22, AZA4 and AZA5 showed an extra OH group in C3 and C23, respectively, were also observed [72,73,74]. AZA6 is reported to be like AZA2 and AZA3 having an extra methyl group similar to AZA2 with the lack of a methyl group in the same position as AZA3. Examination of AZAs in shellfish shows that the main AZAs detected in shellfish are AZA1 and AZA2, while AZA3 is detected at low concentrations [66,67]. AZA4 up to AZA23 (except AZA11) have been shown to be shellfish metabolites, as they have not been detected in planktonic samples [75].

Azaspiracids toxins are fat-soluble polycyclic ethers that are non-neurotoxic in the lipophilic toxins categories. AZAs toxins are heat resistance, acid stable and have a long-term stability under refrigeration [71]. Although AZAs induce some symptoms such as stomach cramp, diarrhoea and nausea they are not categorised as diarrhetic shellfish poisoning (DSP) [76]. In vivo studies, injecting AZA into mice show a neurological action causing paralysis and spasm [70]. Abal et al. studied the effect of AZAs on Caco-2 cell lines, by passing AZA1 through the cell monolayer and cell alterations [76]. In addition, human breast cancer cell line (MCF-7) was exposed to AZA1. The results indicated that AZA1 induced selective cell death [77].

The lethal dose of AZAs injected into mice is reported as 200 µg/kg for AZA1, 110 µg/kg for AZA2, 140 µg/kg for AZA3, 470 µg/kg for AZA4 and 1000 µg/kg for AZA5. However, the lethal dose of oral fed AZA1 in mice is reported as >700 µg/kg (Table 1) [78]. AZAs are produced by *Azadinium spinosum* and *Amphidoma languida* [71] and they have a regulatory testing limit of 160 µg azaspiracids per kg (bivalve tissue) reported by the European Commission due to their multi-organ toxicity [28]. Calcium, adenosine 3,5-cyclic monophosphate (cAMP), Protein kinase C (PKC), phosphodiesterases (PDEs) and mitochondria play roles in mechanism of action of AZAs [52]. Table 1 shows the highest permitted level of marine biotoxins in total quantities (measured in the whole bivalve body or any separate edible part) by the European Commission [28].

**Table 1:** Maximum permitted level of marine biotoxins in total quantities. “Total quantity means measured in the whole bivalve molluscs body or each edible parts separately” [28].

Biotoxin	Limited Level
Paralytic Shellfish Poison (PSP)	<800 microgram/kg
Amnesic Shellfish Poison (ASP)	<20 milligrams of domoic acid/kg
okadaic acid (OA), dinophysistoxins and pectenotoxins together	<160 micrograms of OA equivalents/kg
Yessotoxins (YTXs)	<3.75 milligram of YTX equivalent/kg
Saxitoxin (STXs)	≤800 µg STX.2HCL equivalent/kg
Azaspiracids (AZAs)	<160 micrograms of AZA equivalents/kg
Domoic acid (DA)	<20 mg domoic acid/kg
Brevetoxin	<200 mouse units or equivalent
Ciguatoxins	<0.1 µg/kg fish

### 2.2. Hydrophilic Biotoxins

Hydrophilic biotoxins (domoic acids and saxitoxin) are naturally occurring toxins that can be found in phytoplankton and in the shellfish that consume them. They are classified into two groups according to the type of illness associated with ingestion: amnesic shellfish poisoning (ASP) and paralytic shellfish poison (PSP) respectively [79].

#### 2.2.1. Domoic Acid

Amnesic shellfish poisoning (ASP) was first detected in 1987 in Canada, the toxin responsible was domoic acid (DA), an amino acid of kainoid class produced by phytoplankton. DA can bioaccumulate in shellfish and thus can be introduced into the human food chain (Table 2) [80]. DA can contaminate shellfish such as mussels, crabs, scallops, razor clams and cockles in which it is primarily located in digestive glands [81]. Domoic acid is a heterocyclic amino acid that includes a proline ring and imino group in the structure. In addition, DA consists of three carboxylic acids (Supplementary Figure S1d) [82]. Epi-domoic acid (epi-DA) and isodomoic acids A-H (iso-Das) are isomers of DA that can co-occur in shellfish [27]. These toxins cause gastrointestinal and neurological symptoms after the consumption of contaminated seafood. Although DA is heat resistant and stable at cooking temperatures, there is the possibility of concentration reduction due to the hydrophilicity (water solubility) of the toxin [27]. In vivo studies showed that DA absorption is very low through the gut, the detected amount of DA in animals fed DA showed that rats and cynomolgus monkeys expelled 2% and 4–7% DA, respectively, in their urine after 24 h [83]. Further, DA has a LD_50_ of 2.4 mg domoic acid/kg b.w., the LD_50_ was established by injecting DA extracted from mussels into mice, this induced neurological symptoms including memory loss. Abdominal cramp was also observed [84]. The injection of 1 mg/kg b.w. domoic acid into rodents shows that DA stimulates the thyroid hormones [85]. The mechanism of neural dysfunctionality refers to the tendency of DA binding to glutamate and kainate receptors in the brain. This strong association is due to the similarity of DA to glutamic acid and kainic acid [86]. According to the regulations, the maximum concentration of DA in shellfish should not exceed 20 µg/g (20 mg/kg), otherwise gastrointestinal and neurological symptoms such as vomiting, diarrhoea abdominal cramps, confusion and seizures may result (Table 1) [20].

#### 2.2.2. Saxitoxin (STX)

Saxitoxin (STX) and gonyautoxins are two potent biotoxins produced by Harmful Algae Blooms (HABs) such as red tides, these toxins are found in algae spices, mostly in *Alexandrium* dinoflagellates (Table 2) [87]. STX is primarily related to marine dinoflagellates (eukaryotes) and freshwater cyanobacteria (prokaryotes) [88]. A trait of *Alexandrium* spp. is that they can be found in the same place for several years as their cysts can reside and reproduce in marine sludge [87]. STX has three rings in the structure (Supplementary Figure S1e) and can be described as a 3,4,6-trialkyl tetrahydropurine (Supplementary Figure S1d) with a ring containing a hydrated ketone created through the 3,4 positions of the purine ring. In addition, STX has two guanidine moieties that form by NH_2_ group in 2,8 position of the purine ring [89,90].

STX and its derivatives are often called paralytic shellfish toxins (PST) and cause paralytic shellfish poisoning (PSP) [27]. Neurological symptoms of these toxins can appear shortly after contaminated shellfish is consumed which can cause muscular paralysis and respiratory failure by obstructing voltage-dependent sodium channels resulting in death, a few hours after the first symptoms is observed [91,92]. The lethal dose of STX based on mouse bioassay is 10 µg/g b.w.; however, the oral dose for human is 7 µg/kg b.w. (Table 1) [93].

The first STX was reported in 1798 in Canada where those who consumed mussels became ill [94]. STX including 3,4-propinoperhydropurine tricyclic system and two guanidine groups are highly polar [92] amorphous compounds having a pK_a_ value of 8.24 and 11.60 [95]. STX is a water soluble contaminant, however, it is also soluble in methanol and ethanol [92]. The stability of STX is based on the environmental conditions such as pH and temperature. STX is more stable in acidic environments at a pH around 2–4, however at higher pH there is a decrease in the stability [93]. In addition, suitable temperatures for STX stability is reported at around 20 °C, therefore a pH (2–7) and a temperature of 20–25 °C, are the most suitable environmental conditions for STX and its derivatives’ to remain stable [96].

### 2.3. Emerging Biotoxins

This category of toxins have distinctive differences to the known toxins, as they might be found in the water based on changes caused to the environment such as dinoflagellate cysts discharges, ballast water from ship discharge, red tides, climate change and changes to ocean temperature currents [3,97].

#### 2.3.1. Brevetoxins

Neurotoxic Shellfish Poisoning (NSP) is a gastrointestinal and neurological disease caused by molluscs and shellfish contaminated with brevetoxins (PbTxs; Table 2) produced by the dinoflagellate, *Karenia brevis* [98]. Blooms [20] mainly occur in the Gulf of Mexico and off New Zealand coasts [97]. Brevetoxins are potent cyclic polyether compounds and have nine analogues (Supplementary Figure S1f). Depending on the ether rings there are two type of backbone structure for brevetoxins including type-A 10 and type-B 11 trans-fused ether rings. Among brevetoxin derivatives, PbTX1 is the most potent and PbTX2 is the most naturally abundant in algal blooms [99,100], PbTX3 is the most abundant in beachside marine aerosols [101]. PbTX1, PbTX2, PbTX3 (Supplementary Figure S1f–h) and PbTX7 have an A-type backbone (open lactone ring) and show greater polarity, compared with close-ring brevetoxin derivatives [101]. Brevetoxins are fat-soluble neurotoxins that can cause extensive fatality in marine mammalians and fish [99]. Brevetoxins are highly stable under conditions such as dry state and over the pH scale, ranging between pH 2 and pH 10. Brevetoxins are stable in solvents such as DMSO, alcohol, acetonitrile and acetone [100] but its degradation by incubating at 500 °C for 10–15 min has been reported [102]. Vomiting, diarrhoea, hypotension, arthralgia, myalgia, hyporeflexia are symptoms of NSP [20] additionally, NSP can cause neurological symptoms, respiratory irritation and chest pain. Symptoms of neurological illness persist for longer periods in patients compared to gastrointestinal symptoms [97]. Brevetoxins can cause damage in nerve membranes by influencing and activating voltage-gated sodium channels (VGSC), depolarizing the nerve membranes causing neuro-excitation that leads to neurological symptoms [97]. Moreover, studies have revealed that muscle depolarization and nerve depolarization can happen at the same time [103]. In vivo studies injecting brevetoxin in rats showed that due to the lipophilic nature of NSP, it the toxin easily penetrates cell membranes [104]. The lethal oral dose in mice is 0.520 mg/kg body weight and 0.094 mg/kg body weight (Table 1) after injecting PbTx-3 [97].

#### 2.3.2. Ciguatoxins

Ciguatera fish poisoning (CFP) is a fat-soluble toxin accumulated in a range of fish and marine species (Table 2) [3]. CFP is the most frequently detected toxin in seafood affecting human health [105]. This type of toxin can be found mainly in tropical areas however, these biotoxins can remain in frozen food and may be spread to other regions by the importation of fresh or frozen contaminated seafood [106]. These toxins are heat resistance and stable under moderate acidic or basic environment. Ciguatoxins (Supplementary Figure S1i–m) are polyether compounds including 13–14 aromatic rings joined by ethers that create a ladder-like structure [107]. Ciguatoxin (CTX) was found in 1606 in the South Pacific Islands [100]. These toxins are of a class of compounds with 24 associated structures [100]. They can be produced by micro algae such as dinoflagellate that live in shallow tropical waters that attach to dead coral or seaweeds. Therefore, fish grazing on such corals and seaweeds are at risk of contamination with CTX. In addition, accidental consumption of marine in shore or coastal water containing CFPs can cause illness in humans [105]. The CTX mechanism of action is similar to that of brevetoxin, bonding to sodium-voltage channels that are closed during the resting membrane potential and causing neurological symptoms by depolarization of the membrane of the nerve [105,108]. CTX mostly accumulates in vivo in fish heads, fish livers or fish gonads rather than fish meat in which much lower concentrations are found. More serious poisoning therefore happens after consumption of contaminated fish heads or fish organs [105]. Ciguatera fish poisoning shows neurological [109], gastrointestinal as well as cardiovascular symptoms [107]. Cardiovascular symptoms usually happen in parallel with gastrointestinal or neurological symptoms and patients require immediate medical care [110]. In vivo studies on CTXs shows that these toxins can quickly absorb via the gastrointestinal tract and pass around the body [105]. Symptoms of contamination with CTX include tingling, vomiting, diarrhoea and some neuropsychiatric symptoms such as depression and memory loss [106]. However, neurological symptoms such as confusion, depression [111], loss of memory and anxiety [112] may be observed from one day to weeks after consumption. The lethal dose (LD_50_) is 0.25 µg/kg for P-CTX1, 2.3 µg/kg for P-CTX2 and 0.9 µg/kg for P-CTX3 in mice (Table 1) [113]. Among ciguatoxins, P-CTX1 is the most polar and most toxic form. Indeed, increasing polarity of ciguatoxins in vivo is due to their oxidative metabolism as they climbs up the food chain [107].

**Table 2:** Marine biotoxin classification.

Toxin	Formula	MW (g/mol)	Chemical Class	Syndrome Category	Solubility	Origin	Polarity	Refs.
Okadaic acid (OA)	C_10_H_17_N_7_ O_4_	804	Polyether, spiro-keto ring assembly	DSP	Lipophilic	*Halichondria* *okadaii*	Low polarity	[30,41]
Yessotoxin (YTX)	C_55_H_82_O_21_S_2_	1143.4	Sulfur bearing polyether	Gastrointestinal, Neurological	Lipophilic	*Protoceratium reticulatum, Lingulodinium polyedrum* and *Gonyaulax spinifera*	Highly polar	[50]
Azaspiracids (AZA)	C_47_H_71_NO_12_	842.1	Polyether, second amine, 3-spiro-ring assembly	DSP	Lipophilic	*A. spinosum*	Low polarity	[66,114]
Domoic acid (DA)	C_15_H_21_NO_6_	311	Cyclic amino acid, 3 carboxyilic acid groups	ASP	Hydrophilic	Phytoplankton	Highly polar	[115]
Saxitoxin (STX)	C_10_H_17_N_7_O_4_	299.3	Tetrahydro-purine alkaloid	PSP	Hydrophilic	Phytoplankton	High polarity	[89]
Brevetoxin (PbTxs)	C_49_H_70_O_13_	867.1	Polyether with contiguously fused rings	NSP	Lipophilic	Dinoflagellates	Polar	[99,101]
Ciguatoxins (CTX)	C_60_H_86_O_19_	1111.313	Polyether	Gastrointestinal, Cardiovascular, Neurological	Lipophilic	Dinoflagellate	Polar to moderate polarity	[105]

## 3. Solid Phase Absorption Toxin Tracking

In countries that host indigenous shellfish industries, regular monitoring of algae blooms and biotoxin levels in marine waters are essential public health measures; warnings of imminent or actual contamination events may minimize consumer exposure to potentially harmful shellfish products [116]. Occurrences of harmful algae blooms have dramatically increased in recent decades due to increasing eutrophication and other factors such as the spread of toxins in ballast water discharges [117,118], climate change [119,120], ocean acidification [121], and global shellfish product trading [119]. Alterations to the physical, chemical or biological properties of water environments may cause socioeconomic impacts across multiple sectors (e.g., public health, tourism, recreation and commercial fishing, etc.) in addition to incurring monitoring and management costs [122]. Historically, human health threats posed by biotoxins were identified by direct food product chemical monitoring and by animal testing [75]. Thus far, food tracking programmes are limited to known toxin categories and have not linked toxin occurrences to all HAB populations that give rise to them, so these programmes have no preventative value [19]. Detection techniques based on toxin ingestion by live animals give rise to irreconcilable ethical issues [123] and have very low selectivity therefore limit their effectiveness for protecting consumers [124]. Nevertheless, the Mouse Bioassay (MBA) method developed in the 1970s to detect Lipophilic Shellfish Toxins (LSTs) has until recently remained the reference technique for biotoxin detection, a role now filled by non-animal testing methods especially LC-MS/MS. Nevertheless, animal test regimes can be advantageous: MBA is applicable for new or other type of toxins rather than the known regular biotoxins [75] and cyclic imines that are not categorized in the EU regulations may be detected using the MBA [75]. Rat bioassay (RBA) is specifically used for the detection of OA, DTXs and AZAs and is not commonly used as a monitoring method [75].

**Scheme 1:**
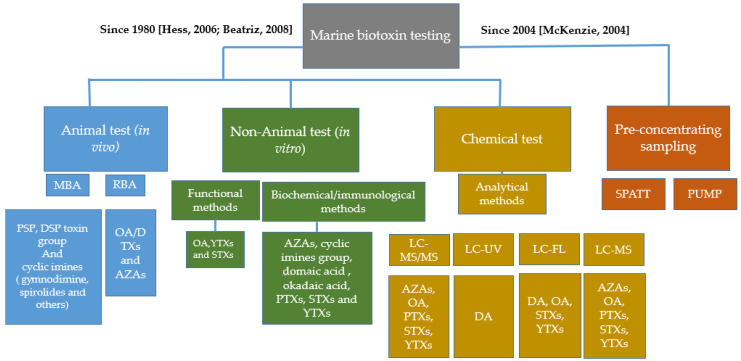
Illustration of marine biotoxin groups and applied analytical techniques. MBA (Mouse Bio Assay) and RBA (Rat Bio Assay).

Mouse bioassay methods only present qualitative (negative or positive) results and combination with and interference from other compounds during sample preparation gives rise to high risk of false positive or negative results [125]. Furthermore, the use of live animals for bioassays raises ethical issues [123]. These test methods are insensitive therefore their capability to protect consumers is limited [124]. Animal testing has been superseded by liquid chromatography–mass spectrometry (LC-MS/MS) [47,67,126,127,128] and high resolution mass spectrometry methods that are able to detect both qualitatively and quantitatively the different toxin classes OA/DTXs, PTXs, YTXs, AZAs and emerging biotoxins either individually or in combination (Scheme 1) [75,129].

Functional assays, methods of detecting toxins based on observable or measurable responses of cells within cell cultures to biotoxins in sample water have been reported by Rossini et al. [130] for neuro-2a neuroblastoma system cell line for STXs and Jellett et al. [131] for a BE(2)-M17 neuroblastoma cell line for STXs; Nicholson et al. [132] for cell-free cell line for okadaic acid and Vieytes et al. [133] for a MCF-7 cell line for YTX. However, it must be noted that cell-based methods are limited to specific toxin groups [19].

Biochemical methods are based on the bonding of marine biotoxins to antibodies taken from animals or cell cultures. The advantage of this method is that they are suitable for the detection of very low concentrations of specific toxins, including AZAs [75], the cyclic imines group [134], domoic acid [135], okadaic acid [136], PTXs [137], STXs [131] and YTXs [138]. The commonest chemical analysis methods employed for detecting marine biotoxins are based on liquid chromatography (LC), in tandem with other physicochemical methods such LC-MS, LC-UV and LC-FL [75]. LC-MS/MS is the official method for lipophilic toxin detection [139], HPLC-UV for domoic acid detection [140], HPLC-FLD for PST toxin detection. Emerging toxins and other toxins are primarily analysed using LC-MS/MS and with biological functional methods [141,142,143,144,145]. The application of LC with fluorescent detection (FL) [146,147], LC-MS [139,148,149] and LC-MS/MS [148] has been widely applied and evaluated for detection of the DSP group of toxins; LC-MS [149] and LC-MS/MS [150] for PTXs; LC-MS/MS [151] and LC-FL [152] for STXs and LC-FL [153], LC-MS [149] and LC-MS/MS [151] for detection of YTXs (Scheme 1). Applying analytical methods for the detection of marine toxins is expensive [154], requires extensive validation and highly trained staff for instrument operation but analytical methods are the most dependable and reliable analytical approaches for protecting human health [75,155].

The foregoing discussion suggests a need for cost-effective but efficient methods to replace discrete grab (non-continuous) sampling traditionally employed to monitor marine biotoxins. However, concentration of toxins present is an important requirement in these sampling methods and sampling might not capture spatial concentrations due to vertical migration, hydrologic or circulation effects, therefore results might be inherently biased [156,157].

The concept of SPATT (small packages of adsorbent phase deployed for extended durations at various depths in the water column) was pioneered by McKenzie et al., an idea that first arose from observations of dissolved polar and non-polar biotoxins in seawater in 1998 and from culture studies conducted in 2003. Passive adsorption followed with LC-MS detection gives rise to a fast but simple and sensitive biotoxin monitoring system that McKenzie et al. concluded could provide accurate predictions of net toxin accumulation by bivalves (Supplementary Figures S2 and S3). The technique was first field tested in 2002 and 2003, a period that included DSP and YTX bloom events (Supplementary Figure S4) [116,158].

SPATT technology, in common with other continuous monitoring techniques, can be directly deployed in the marine and freshwater environment, an advantage over traditional lab-based methods such as shellfish tissue testing or phytoplankton microscopic cell counting. In addition, SPATT technology is a simple, cost effective technique for recovering targeted analytes from a aqueous environment [159]. However, SPATT will only detect dissolved biotoxins (extracellular biotoxins) which is one of its limitations, another drawback is the lack of calibration and validation for this technique [19]. Furthermore, the SPATT technique is not able to detect at the ng/g of toxin concentration required by health advisory authorities [159]. However, it is still a useful predictive and preventative tool for toxin monitoring when used in conjunction with analytical instrumentation for the screening of seafood.

### 3.1. SPATT Bag Construction and Adsorbent Phase Activation

SPATT bag dimensions are typically 60 mm × 60 mm, bags being fabricated from 95 µm polyester mesh (Polymon PES 95/27). Edges are closed by polyester thread stitching [116] or by heat-sealing. Supplementary Figure S2 illustrates construction of SPATT bags [160].

To form a thin layer of resin Rundberget et al., placed the resin between two layers of nylon mesh that was clamped tightly into a frame (Supplementary Figure S2) [161].

Another example (Supplementary Figure S2) illustrates SPATT bag and associated mounting system construction. For this river deployment, bags were secured to a large aluminium alloy mounting tube using 4 mm diameter clamping screws [162].

### 3.2. SPATT Sorbents

A number of synthetic adsorbent materials show affinity for biotoxins. In common with naturally occurring counterparts (e.g., Zeolytes and activated carbon), human-made sorbents are characterized by spherical particles with large surface areas and high porosities that create three-dimensional crystalline lattices, including pores, in which water molecules are held loosely. Adsorption results from interactions between atoms at the sorbent surface [163].

While sorbent particles are in contact with a solution, smaller solutes can diffuse into pores using physical interactions between the surface and the species. The precise nature and combination of bonds include non-polar hydrophobic interactions, polar interactions (h-bonding, pi–pi bonds, dipole–dipole or induced dipole), electrostatic attraction; and depend on analyte species. Adsorption and desorption occur simultaneously according to differential concentrations of analyte in solution and in the sorbent, giving rise to characteristic analyte-specific behaviour indicated in Figure 1 in Section 1. The complex nature of interactions necessitates an experimental approach to determining mathematical models that best fit observed isotherms. Studies such as Shin and Kim [163] demonstrate this principle; however, searches for evidence of similar research into SPATT sorbent biotoxin affinities at the time this review was prepared were unsuccessful. Larger molecules, those that are bigger than pore size, will remain unabsorbed, a phenomenon known as the ‘sieving’ effect.

Synthetic adsorbents have a wide range of applications in the separation of valuable compounds from plant extracts, fermentation products, food additives and in pharmaceutical applications. Synthetic sorbents are designed to remain stable in acidic and alkaline environments and are resistance to organic solvents. Analyte extraction from synthetic sorbents is safer and reduces solvent usage compared to other solvent-based extraction techniques.

Synthetic sorbents can be categorised into three different groups based on their chemical structures:

#### 3.2.1. Aromatic Adsorbents

Aromatic adsorbents are characterized by a crossed linked polymeric matrix that are suitable for the separation of peptides, antibiotics and food additives [164], for example, the type of commercial sorbents of this type are DIAION HP20, HP21 SEPABEADS SP825L, SP850, SP70 and SP700. DIAION HP20 is the most popular non-polar aromatic-based synthetic sorbent with particle density of 1.01 g/mL, specific surface area of 600 m^2^/g and large pore size (200–300 Angstrom), which enables adsorption of large natural products and organic compounds with molecular mass > 1000 Da, such as peptides, proteins and phenols [164]. This sorbent can adsorb many lipophilic marine toxins and some hydrophilic toxins (see Section 3.4) and has exhibited the reliable adsorption and recovery of the freshwater hepatotoxins, microcystin (MC) MC-LR, MC-YR, MC-LA, and MC-RR [165]. SEPABEADS SP825L and SP850 aromatic sorbents are similar to HP20 having high porosity however, they have higher surface areas and a more uniform pore-size distribution compared to HP20 (Supplementary Figure S3a) [164].

SEPABEADS SP700 (Supplementary Figure S3b) has also been reported as an effective lipophilic shellfish toxin detection sorbent. SP700 pore size is smaller than that of HP20 with pore radius of 90 Å [166]. SP700 provide greater surface area (1100 m^2^/g), with similar particle density (1.02 g/mL) (Supplementary Figure S3b) [167].

SF700 has applications in the separation of food additives and in food purification (refining impurities and unwanted products from food ingredients) of chemicals. In addition, SP700 are able to adsorb vitamins, antibiotics, enzyme and steroids from aqueous environment [167].

#### 3.2.2. Modified Aromatic Adsorbents

Modified aromatic adsorbents are designed to give the highest hydrophobicity (water repellent). Modification by brominating the aromatic region enables this phase to adsorb dissolved components or those components existing in a low concentration in an aqueous solution. An example of a modified resins is SEPABEAD SP207 (Supplementary Figure S3c), which has a 1.18 g/mL particle density, particle size > 250 µm, and it is suitable for the adsorption of organic compounds at very low concentrations. Furthermore, being a hydrophobic sorbent with high selectivity for non-polar molecules, this material is suitable both in applications with upward flow (fixed bed processes) and in batch processes [166].

#### 3.2.3. Methacrylic Adsorbents

Methacrylic series are methacrylic ester copolymers with a highly hydrophilic nature, suitable for adsorption of polyphenols and surfactants. DIAION HP2MG belongs to this group of sorbents. DIAION HP2MG is synthesised from methacrylate and has no aromatic compounds in its chemical structure (Supplementary Figure S3d). This polymer resin is of intermediate polarity and has more hydrophilic specifications in its matrix, this characteristic enables it to be used in desalting and in adsorption of high polarity organic compounds [166].

#### 3.2.4. Other SPATT Adsorbents

Other SPATT adsorbents include AG 50W-X4 (strong Cation exchange) and Amberlite IRP-64 (weak Cation exchange) phases which both have been shown to adsorb the polar cyanotoxin anatoxin-a [168]. HLB (Hydrophilic-lipophilic polymeric resin) show a high adsorption capability for freshwater hepatotoxins microcystins (MC), MC-RR and MC-LR [6].

#### 3.2.5. SPATT Sorbent Comparisons

Since SPATT bags were first developed, numerous studies have assessed the efficiency of adsorbing a range of aquatic toxins (i.e., amount recovered compared to concentration in environmental water) [116]. Table 5 shows an overview of the main results from in vitro and in situ studies conducted on SPATT technology and different sorbent substrate types for monitoring microalgae and cyanobacteria in marine and freshwater environments.

DIAION HP20 is inexpensive (approximately EUR 200/kg), widely used and has been tested for collecting a variety of lipophilic and hydrophilic toxins. Zendong et al. [154] illustrated HP20 resins use in SPATT bags and determined a strong correlation between toxins extracted from SPATT devices and toxins levels in phytoplankton rich aqueous solutions. This study also investigated the amounts of OA, DTX1, PTX2 accumulating according to resin mass (0.3, 3 and 10 g), concluding that 3.0 g of sorbent had better extraction results compared to 0.3 g but the larger 10 g quantity was not recommended because of the risk of clogging.

Caillaud et al. [169] reported 89% and 66.2% recovery efficiencies of ciguatoxins (CTX1B) and maitotoxins (MTX), respectively, from 10 g of wet DIAION HP20 resin after 72-hrs exposure. Amounts of 4, 2.8, 0.3, 0.4, 0.5, 0.2 ng/g HP20 resin were detected for OA, YTX, AZA1, PTX2, GYM and SPX-13-DesMe-C, respectively.

A study conducted by Fux et al. [5] off the Irish coast reported 36% and 62% recovery efficiency of OA, DTX1, PTX2 and YTX from DIAION SP700 and HP2MG, respectively. PTX2, OA and YTX showed 50%, 60% and 80% extraction ability, respectively, from the HP20 resin [116].

Lane et al. [170] applied HP20, SP700, SP207, SP207SS to detect DA and PST. All resin sorbents were successful in extracting DA although the HP20 was reported as the most effective. For lipophobic biotoxins, the efficiency order was reported as: HP20 > SP700 > SP207 > SP207SS.

Oasis HLB, Strata-X and HP20 resins were tested alongside low-density polyethylene strips and silicone rubber strips for the detection of OA, AZAs, PnTX-G, SPX1, PITXs in laboratory and field tests [2]. Polymeric strips yielded significantly lower recoveries than sorbents, and the toxins of all groups were absorbed more slowly by HP20 when compared to Strata-X and Oasis HLB. All sorbents showed different efficiencies and accumulation speeds with Strata-X and Oasis HLB proving to be more suitable for short-term deployments or in-field evaluation of toxin presence, but HP20 was more appropriate for exposure periods of greater than 5 days.

SPATT bags containing PAC and Strata-X were deployed in a river containing toxic benthic cyanobacterial mats by Wood et al. [162]. Powdered activated carbon (PAC), a cheap, widely available sorbent that slightly outperformed Strata-X in tests, with anatoxin-a (ATX) and homoanatoxin-a (HTX) toxin substrates, giving 45% desorption recovery compared to 42% recovery from Strata-X.

SP700 and HP20 have been compared in several studies [171]. SP700 showed more rapid short-term accumulation but no significant difference after 72 h exposure. However, Fux et al. [172] found HP20 did not reach equilibrium in that time frame (72 h), but in long-term field trials HP20 showed significantly greater adsorption potential than other sorbent resins such as SP700. Other researchers, taking desorption into account, found that the lower recovery efficiency of SP700 (78% and 72% for MC-LR and [Dha7] MC-LR freshwater toxins, respectively, compared to 91% and 89% for HP20) offset speed of adsorption and recommended HP20 for toxin tracking [173]. Adsorption data analysed by Li et al. [174] indicated SP700 has a lower capacity for OA and DSP (1088 and 1872 g/g) toxins than HP20 (1639 and 2934 g/g), respectively, and that adsorption capacity is determined primarily by pore size distribution and analyte polarity, rather than sorbent specific surface area [174].

### 3.3. SPATT Bag Preparation and Analyte Extraction

SPATT bags are typically prepared in the following steps: (i) cleaning, then rinsing, with deionized water, (ii) activation by exposure to 100% MeOH, (iii) dispersal of specific volumes into plankton net or nylon mesh and sealing. The activation and dispersal sequence varies in different studies, for instance dry resin can be dispersed through the mesh before the activation process [170].

Synthetic sorbents are spherical cross-linked polymers, that due to this specific structure, show noticeable swelling in the presence of polar solvents such as methanol and water [175]. Extraction of toxin substrates from a resin sorbent involves several steps [159], and with different eluates as outlined in Table 3. The choice of eluates varies based on the type of targeted toxin. For example, with toxins PTX, PTX-2 SA, PTX-11, PTX-11 SA, OA, OA-ester or YTX, the optimised elution solvent is 100% MeOH [2,156]. In addition, DA and CTXs elution was reported as requiring three different elutes in each step starting with MeOH in water, followed up with ammonium acetate in MeOH for the last two steps [176] (Table 3 and Table 4).

### 3.4. SPATT Bag Storage and Stability

Methods for SPATT bag storage reported in various studies typically focus on stability issues. It is necessary for bags to remain hydrated before and after deployment, therefore researchers commonly report storing them in deionized water at 4–6 °C prior to deployment [159]. In addition, after deployment, the SPATT bags are required to be soaked in the elution solvent (elute) immediately or be kept in storage at −20 °C [159]. SPATT bags stored in the freezer (−20 °C) were stable with no loss of biotoxins for up to three months; to-date no longer freezer (−20 °C) storage duration has been assessed. In addition, under the same conditions there are no sign of degradation of extracts up to 12 months [6,165]. The advantages of the SPATT monitoring include:

(i) simplicity, low cost, ease of application, transport and storage [160];
(ii) allows sampling throughout the water column where no shellfish exist naturally [19];
(iii) targeting toxin substances directly [19,160];
(iv) impervious to biotransformation with no sign of degradation when stored in −20 °C [160];
(v) a sufficient pre-concentration technique to ensure adequate adsorption and analytical detection;
(vi) can be used as an early warning system for bloom events when coupled with appropriate analysis (e.g., ELISA, LC-MS) [160];
(vii) reveals unique information on toxins such as origin, environmental durability, and variations in the specific toxicity [6,160];
(viii) profiles the water for toxins generated by HABs prior to their biochemical transformations within shellfish tissues that leads to a variety of toxin derivatives and
(ix) assesses biotoxin frequency, and the duration of algae blooms in a specific region [160].

Phytoplankton monitoring disadvantages include:

(i) difficult to detect spatially and temporally integrated water samples [116];
(ii) monitoring only shows the evidence of a possible shellfish contamination [116];
(iii) phytoplankton monitoring is intensive, difficult to identify and needs specifically skilled observers [116].

The difficulties of routine testing of shellfish include:

(i) high cost of instrumentation with training requirements and complex sample preparation and clean-up optimisation and validation processes;
(ii) biochemical transformations within shellfish tissues leads to a variety of toxin derivatives, a more complex toxin profile than what originated from the HABs and
(iii) matrix problems from biological samples makes the extraction and analysis slow, and sometimes analytically challenging [2,5].

### 3.5. SPATT Applications

#### 3.5.1. Application of SPATT to Marine and Freshwater Toxins

Solid Phase Adsorbent and Toxin Tracking (SPATT) is a powerful, reliable and efficient biotoxin monitoring tool applicable to both marine and freshwater environments. Harmful Algae Blooms (HAB) can produce toxins that cause illness or fatalities following consumption of contaminated shellfish and seafood [47], however, the amount of toxin accumulated in shellfish varies based on toxin production and growth stage so the presence of toxin does not necessarily mean that shellfish are a contamination risk [165]. HAB proliferation has resulted from eutrophication, unregulated ballast water discharges and climate change. Total toxin regulatory limits for marine biotoxin in seafood has been set by EC No 853/2004 regulations [28]. The SPATT tool coupled with a laboratory based analytical approach such as LC-MS/MS provides a selective and highly sensitive tool for detecting biotoxins. Table 5 (Marine and Fresh water applications) reviews the use of SPATT tool in monitoring toxin-producing algal, results indicate that SPATT performs well for monitoring dissolved polar (domoic acid) and non-polar (polyether) biotoxins species and SPATT can also be used as an early warning, forecasting system [116].

The first SPATT bag was designed by MacKenzie et al. in 2004 to detect dissolved biotoxins during diarrhetic shellfish poisoning (DSP) and yessotoxin (YTX) blooms caused by *Dinophysis acuminate* and *Protoceratium reticulatum*, respectively. Such an early warning method may predict the net accumulation of polyether marine biotoxins in mussels. HP20 with a 3 g dry weight (12 mL in solution) sorbent was placed and sealed in the 95 µm polyester bags and conditioned with MeOH and rinsed with MQ-water in advance of deployment and bags deployed at different depths to track cumulative adsorption within the water column. The extraction process performed after initially washing the retrieved SPATT with ultrapure water (Milli-Q water) to remove salt residues, involved removing the phase from the SPATT bag and transferring the phase to an empty solid phase extraction (SPE) tube with a glass filter. The adsorbent phase is eluted with 100% MeOH. Recoveries were determined by LC-MS/MS [116].

Fux et al. studied the uptake and extraction behaviour of OA and DTX1 applying five different sorbents, DIAION HP-20, SP850, Sepabeads1 SP825L, Amberlite1 XAD4, Dowex1 Optipore1 L-493. Results demonstrated that HP20, SP850 and SP825L are similar in adsorbing the lipophilic marine biotoxins with recoveries of 99%, 97% and 98%, respectively [172]. In addition, Fux et al. applied HP20 to the adsorption of numerous lipophilic toxins and demonstrated that SPATT disks are sensitive tools for toxin profile investigations and suggested that the OA toxin groups in the absence of toxic phytoplankton did not result in shellfish contamination. It means that feeding bivalves on toxic phytoplankton enhance the accumulation of toxins such as the OA or DTX group [177]. Furthermore, SEPABEADS SP825L, SP850 & SP700 were applied in the adsorption studies of OA, PTX2, AZA and YTX [160]. Recoveries of SP700 for OA (61%), PTX2 (22%), AZA (41%), YTX (47%) was reported by MacKenzie [160]; HP20, SP825 and SP850 showed similar recovery results. HP20 having a larger pore size could not reach the equilibrium within 72 hrs as achieved for sorbents SP850 and SP825L. Fux et, al. related this to the diffusion concept film diffusion, that is the migration of molecules to the surface of the particles and internal diffusion that is the migration from the surface to the internal part of the resin particles [172]. SPATT was used to determine recovery characteristics of MC-LR, MC-YR, MC-LA and MC-RR in several studies [178]. The recovery results showed the suitability of SPATT for monitoring microcystin (MC) toxins in freshwater and marine environments [157,158,179]. Stata-X polymeric resin was successfully used in SPATT bags for monitoring cyanobacteria, ATX and HTX, in river waters [162]. A comparison study on individual HP20, Strata-X, BundEly C18 and Oasis HLB SPATT bags showed that the adsorption rate on Oasis HLB and Strata-X were higher than on HP20, although HP20 and Strata-X gave a higher recovery after 24 h of exposure [2].

**Table 5:** Marine and Freshwater applications.

SPATT Resins	Toxins Detected	Year	Country	Elute	Application	Deployment Condition	Adsorbent Quantity	Analyte % Recoveries	Ref.
**DIAION HP-20** (**Bags**)	PTX, PTX2 SA, PTX11, PT11 SA, OA, OA-ester & YTX.	2004	NZ	MeOH Ace MeOH > Ace	Marine	Deployed at selected depth	12 mL = 3 g dry weight	Ave. = 62%	[116]
**DIAION SP-207** (**Bags**)	OA, DTX1, PTX2, YTX, 36% less recovery than DIAION HP-20	2004	NZ	MeOH Ace MeOH > Ace	Marine	Deployed at selected depth	12 mL = 3 g dry weight	Ave. = 36%	[116]
**DIAION HP-20** (**Large scale pumping**)	OA DTX-2 PTX-2 PTX-2SA	2007	Norway	MeOH	Marine	Seawater	0.5 kg/column	DTX-2: 73% OA: 78% Accumulation: 2.7 mg OA, 1.3 mg DTX-2 and 1.8 mg PTX-2 during an 18-h	[161]
**DIAION HP-20, SP850, Sepabeads1 SP825L, Amberlite1 XAD4, Dowex1 Optipore1 L-493** (**Bags & Disks**)	OA, DTX2, PTX2, AZA1, -2 and -3	2008	Ireland	MeOH	Marine	Deployed	3 g	OA and DTX1 were determined in positive ionisation mode	[172]
**Membrane (Polycarbonate, polyethersulfone, polyester, nylon) and POCIS Oasis HLB**	MC-RR, MC- LR	2008	Czech Republic	90:10 *v*/*v* MeOH/water Acidified with 0.1% TFA	Fresh water	Exposed in a natural reservoir	Membrane exchanging area: 47.5 cm^2^, Oasis HLB: 2.75, 5.55, 11.1 mg/cm^2^	MC-RR: 0.022 L/day, MC-LR: 0.017 L/day	[168]
**POCIS Oasis HLB**	29 organic chemicals: Antibiotics, fungicides, herbicides, biocides	2009	Spain	MeOH acidified in three different levels	Marine	Fish farm	200 mg	The detected conc’s do not have impact on aquatic organism	[179]
**Diaion HP20** (**disk**)	*D. acuta* bloom	2009	Spain	MeOH	Marine	Deployed at different depth	3 g	Plankton: PTX2 ranged from 19–73 pg/cell	[180]
**DIAION HP-20** (**Disks**)	OA, PTX, YTX & AZA group	2009	Ireland	MeOH	Marine	Deployed at different depth	3 g	Accumulation rate of toxins in the mussels and SPATT discs correlated	[177]
**DIAION HP-20** (**Disks**)	20-methylSPX-G, AZA-1, AZA-2, OA, DTX-1, DTX-2, PTX-2, PTX-12 & YTX.	2009	Norway	MeOH	Marine and Fresh water	Deployed attaching to a fixed point (1 m depth)	3 g	PTX-2: 5–40 ng/disk 20-methylSPX-G: 706.5 ng/disk SPX-C: 164.2 ng/disk	[181]
**SEPABEADS SP825L, SP850 & SP700** (**Bags**)	OA, PTX2, AZA, YTX	2010	NZ	MeOH	Marine	Deployed	-	OA: 61% PTX2: 22% AZA: 41% YTX: 47%	[160]
**DIAION HP-20** (**Disks**)	OA, PTX, YTX and AZA group	2010	Ireland	MeOH	Marine (Deployed in four different depth)	Deployed Different depth	3 g	Recovery discussed based on period and depth	[5]
**HP20, SP700, SP207, SP207SS**	Domoic acid and saxitoxin	2010	USA	MeOH	Coastal	Deployed	3 g	SP700: 69–72% HP20: 99%	[170]
**DIAON HP20**	MCY-RR and -LR,	2010	USA	MeOH	Freshwater and Lake water	Deployed	3 g	2.9 million ppb	[157]
**Strata-X** (**Bags**)	ATX, HTX, Dihydroanatoxin-a, Dihydrohomotoxin-a	2011	NZ	MeOH	Freshwater	River 1.2 m^3^/s	1 g	7% of water loading	[162]
**PAC G-60** (**Bags**)	ATX, HTX, Dihydroanatoxin-a, Dihydrohomotoxin-a	2011	NZ	5% FA and 70% MeOH	Freshwater	River 1.2 m^3^/s	1 g	4–12% of water loading	[162]
**Diaion HP20**	CTX, MTX	2011	Spain	MeOH	Marine *G. Pacificus* culture	In vitro experiment	10 g wet	CTX1B: 85.5–90.9% MTX: 66.2%	[169]
**SP700**	PSP toxins	2010	Spain	MeOH	Marine	PSP and LSTs producing culture	1 g	GTX2,3: 406.02 ± 13.30 ng/g resin STX: 219.02 ± 37.71 ng/g resin	[182]
**DIAION HP-20** (**Disks**)	Spirolide C, iso-spirolide C,13-desmethylspirolide C, 20-methylspirolide G	2011	Norway	MeOH	Marine	Deployed	3 g	Spirolide C: 69%, iso-spirolide C: 13%, 13-desmethylspirolide C: 22% 20-methylspirolide G: 77% 13,19-didesmethylspirolide C: 33%	[183]
**SEPABEADS SP700**	Toxic Alexandrium okadaic acid, 13-desmethyl SPX C, 20-methyl SPX G	2011	Ireland	MeOH	Harbour	Deployed in water	5 g	OA, DTX-2 and PTX: 2.5 ng/g	[184]
**HP20**	Pinnatoxin (PnTx), analogues PnTx-E, PnTx-F, okadaic acid (OA) and its esters	2011	NZ	MeOH	Marine	Deployed over two summers	4 g	OA: 14% PTXs: 50% OA-esters: 10%	[185]
**DIAION HP20**	MC-LR, -YR, -LA, and -RR	2011	USA	MeOH	Freshwater	Deployed for 16 months	3 g	MC-LR: 66.4 ng/L, 18,400 ng/g resin equal parts MC-RR, MC-YR, MC-LR	[156]
**DIAION HP20**	Chlorophyll-a, Secchi depth, total phosphorus and total nitrogen	2012	NZ	MeOH	Lake	Deployed	3 g	CYN82/91, CYN83/87/95) and the *Calothrix* sp. (CYN100) had low similarities (<94%) to GenBank sequences	[186]
**HP20 and SP700**	Cyanobacterial cultures	2013	China	MeOH	Freshwater *M. aeruginosa* cultures	Deployed	2 g	HP20 better result than SP700	[173]
**HP20**	OA, DTX2, PTX2)	2013	Spain	MeOH	Marine	Deployed at 3, 7 and 12 m depths	2.5 g	OA/DTX2: 1.5–6.0 ng PTX2: 1.8–7.0 ng PTX2SA: 0.5–3.0 ng	[187]
**Diaion HP20, Strata-X, Oasis HLB, BondElut C18**	SPX1, AZA1, PnTX-G, ovTX-a	2014	France	MeOH	Seawater Agilent reservoir	Conditioning method the same as Shea et al. 2006	Oasis (30 mg), Strata-X (200 mg), HP20 (200 mg), Bond Elut C18 (200 mg)	SPX1: 14 ng, AZA1: 19 ng, PnTX-G: 238 ng, ovTX-a: 359 ng	[2]
**HP20** **XAD761**	DSP toxins	2014	Ireland	MeOH: Water 80:20	Marine	Deployed at different depth	5 g of each separately	HP20 XAD761	[165]
**DIAON HP20**	Cyclic imines (SPXs, PnTXs, GYMs)	2014	Spain	MeOH	Marine	Deployed	10 g wet	DIAON HP20	[188]
**DIAION HP-20 resin**	Microcystin-LR, RR, YR, LA	2014	US	MeOH	Freshwater	Deployed blow surface water	3 g	DIAION HP-20 resin	[189]
**HP20**	A range of lipophilic toxins	2015	France	MeOH	Marine	Deployed	300 mg	HP20	[190]
**Amberlite XAD761, HP20**	OA, PTX2, DTX2	2015	Ireland	80:20 MeOH: Water	Marine	Deployed in different depth	5 g dry weight	Amberlite XAD761, HP20	[6]
**Diaion HP20**	OA, DTX1, DTX2, PTX2	2016	Spain	MeOH	Marine	Deployed in semi enclosed river at 3 m depth	10 g	OA 17.75 pg/cell PTX2 13.2 pg/cell DTX1 trace amount	[191]
**HP20**	PTX2, *D. fortii*, *D. acuminate*, *P. rotundatum*, OA, DTX1	2016	China	MeOH	Marine	Deployed at 8 m depth	3 g	*D. fortii* (0.28 pg/cell), *D. acuminata* complex (0.08 pg/cell) & *P. rotundatum* (*D. rotundata*) (0.02 pg/cell). PTX2 (nd~5.7 mg/kg), OA (nd~2.8 mg/kg) and DTX1 (nd~1.6 mg/kg)	[192]
**HP20**	OA, DTX1, PTX2, PTX2sa, 13-desMe-C, PnTX-G	2016	France	MeOH	Marine	Deployed during summer	0.3, 3, 10 g	The higher amount of resin captured more toxins	[154]
**HP20**	DSTs, AZA	2017	USA	MeOH	Marine	Deployed	3 g	Conc’s during different month are discussed.	[193]
**SPATT** **HP20**	Microcystins	2017	USA	MeOH	Water reservoir and lake	Deployed at different sites	3 g	MC-LR: ~88%, MC-YR: ~100%, MC-LA: ~100%	[178]
**HP20**	Gambierdiscus toxins (CTXs)	2018	France	MeOH	Marine	Deployed	20 g	55 ng P-CTX-3C equiv./g resin	[194]
**DIAION HP20**	DSTs	2018	USA	MeOH & then MeOH: ammonium acetate	Marine	Deployed	3 g	Four toxins were identified in 37% of mussels. one toxin in 99% of mussels	[176]
**Strata-X**	Toxic *Microcoleus autumnalis* (*Basionym Phormidium autumnale*)-dominated	2018	NZ	MeOH acidified with FA	Stream water	Deployed in River 10, 20, 40 m	1 g	0.91 ng mL^−1^ and 95 ng g^−1^ of strata-x hr^−1^	[195]
**HP20** (**Diaion**) **and XAD-2** (**Amberlite**)	OA, STX, DTX1, PTX2, PTX2 isomers	2018	USA	ACN acidified with FA	Marine	Deployed	3 g	For both resins: OA: 53% DTX1: 20% Esterified OA: 19% Esterified DTX1: 8% PTX2: 88% PTX2 isomers: 5% PTX11: 4% secoPTX2: 3%	[196]
**Diaion HP20**	PCTXs, MTXs	2018	NZ	DCM and aqueous MeOH	Marine	In vitro	2.5–10 g	PCTX- 3C (70%) P-CTX-1B (92%). MTX3 not possible to detect	[197]
**HP20**	Domoic acid (DA), saxitoxin (STX), okadaic acid (OA)	2019	USA	Extract 1 50% MeOH (*v*/*v*) and Extract 2 and Extract 3 with 1 M C_2_H_7_NO_2_ in 50% MeOH	Marine	Deployed		DA from 9.2 to 37 ng/g STX from 1.3 to 5.3 ng/g	[198]
**HP20**	Phycotoxin pectenotoxin-2 (PTX2)	2020	Antarctica	MeOH	Marine	Deployed in cove	10 g	Very low background conc.	[199]

Abbreviation: MeOH (Methanol), Formic acid (FA), DCM (Dichlorometane), Ace (Acetone), ACN (Acetonitrile), Ammonia Acetate (C_2_H_7_NO_2_), New Zealand (NZ).

#### 3.5.2. SPATT Sorbents and Biotoxin Harvesting

Rundberget et al. exploited the affinity shown by synthetic adsorbents for biotoxins by developing a large-scale toxin harvesting system, conceived to overcome the problems encountered by toxicology researchers and scientists developing analytical methods in obtaining sufficient quantities of pure toxins and their metabolites [161]. The system comprised novel filter configurations to release toxins from cells while eliminating debris and unwanted insoluble compounds, and a solid phase extraction column of fixed-bed design was used to gather analytes as shown in (Supplementary Figure S4). The system included a submersible pump (A), a pre-filter with 100 µm plankton net (B), 50 mm filter (C), flow distributor (D), columns contain adsorbent resin. On the right-hand side picture of the pre-concentration device, as the second pump has shown, the system passes the water through a 40 µm filter to increase the algal cell concentration (10-fold), the second pump passes the concentrated water to the pumping system. With this apparatus, quantities of okadaic acid and dinophysistoxin-2 were obtained from algal blooms in Spain and Norway, with results indicating that the method was also applicable for collecting toxins such as azaspiracids, pectenotoxins, spirolides and microcystins from in-field sources or microalgal cultures [161].

## 4. Polar Organic Chemical Integrative Sampler (POCIS)

The ‘Polar Organic Chemical Integrative Sampler’ device is a semi-quantitative time integrative sampler or qualitative sampling tool for separating hydrophilic or polar organic compounds with different physicochemical properties from water [200]. POCIS has been using in screening, monitoring and in the determination of dissolved bioavailable contaminants, pharmaceutical residues [201], endocrine disrupting substances [202], pesticides [203], per-fluorinated compounds and mixtures of chemical compounds [204].

POCIS systems comprise one or more analyte receptor capsules directly exposed to environmental water or mounted on a metal frame held in a perforated canister through which water passes freely. Capsules are made up of a sorbent phase component, chosen for its affinity for targeted compounds, trapped between biofouling-resistant microporous polyethersulfone membranes (pore size 0.1 µm). Capsule assemblies are circular or rectangular, secured by stainless steel rings (Supplementary Figure S5). The water matrix, which can include a mixture of dissolved compounds such as pesticides, personal care products and pharmaceuticals [205] can pass through the membrane while chemicals of interest are trapped in the sorbent [206,207]. Total membrane areas are in the order of 41–46 cm^2^ with sorbent masses of 228 mg [205,208], give surface area to mass ratios of around 180 cm^2^/g (membrane diameter 3.3 cm giving surface area 17 cm^2^). Deployment durations reported in different studies range between one week to one month (see Section 1)

Accumulation takes place during three successive steps simultaneously: (i) diffusion of the dissolved compound crossing the water boundary layer, in which the thickness of this boundary layer, varies based on the water flow velocity and turbulence around the sample [205]. The water boundary layer is a result of friction between water and sampler that causes a higher viscosity on the surface of the sampler [209]; (ii) the next step is compound transportation. The transportation can occur through two routes, either through the water-filled pores of the PES membrane or through the membrane polymer itself; (iii) the final step is adsorption, that is penetration of the analyte into sorbent resin [205]. Indeed, the challenge of applying POCIS and understanding the accumulation of different compounds and sampling rates is because of the last two steps. Advantages of this multi-transport method include the ability to measure time-weighted average concentrations of analytes, the ability to detect ultra-trace micropollutants, the capacity to use different membranes, its simple construction and the omission of a high-power demand [210].

Analyte components with log values between (0 to 5) 0 ≤ K_ow_ ≤ 5, in which K_ow_ refers to the ratio (without a unit) of concentration of solute to determine the hydrophobicity (the lower the log K_ow_, the higher solubility of solute in the water) [208], are usually targeted by POCIS sorbents, for example the copolymer poly(divinylbenzene)-co-N-vinylpyrrolidone, [206,207]. Proprietary sorbent mixtures are available for target applications, for example triphasic sorbent admixture included in ‘pest-POCIS’ (10 mg sorbent per POCIS disk) for pesticide screening or ‘pharm-POCIS’ which is adaptable for both pharmaceuticals and pesticides monitoring and shows a higher efficiency for sampling most analytes (Supplementary Figure S5) [207].

### 4.1. POCIS Applications in the Marine Environment

Bioactive pharmaceutical residues, as metabolites or original compounds, that end up in the environment through effluent rejection after treatment from wastewater treatment plants, can accumulate and negatively affect the environment by interacting with marine organisms and exacerbating current environmental stresses such as climate change or eutrophication [211]. The use of Polar Organic Chemical Integrative Sampling (POCIS) as a qualitative and semi-quantitative tool for the analysis and monitoring of pharmaceutical compounds in marine environments has been reported [211,212,213]. The determination of the uptake rate or sampling rate (Rs) of the compounds onto the resin sorbent is important in all POCIS studies. The uptake rate varies for different compounds and metabolites. The parameters that can affect the sampling rate include water flow, amount of resin sorbent, temperature, dissolved organic compounds (DOC) and conductivity [212]. Table 6 shows different sorbents used in POCIS for the adsorption of a variety of compounds such as pharmaceuticals, pesticides and personal care products in the marine environment.

Carbamazepine as a highly prescribed pharmaceutical ingredient is the most frequently detected pharmaceutical compound found in marine environment, it is highly resistance to biodegradation and elimination after water treatment. Buento et al. studied carbamazepine and oxacarbamazapine and their related metabolites in marine environment in the Mediterranean Sea using 200 mg Oasis HLB sorbent, at 21 °C sea water temperature. Average recoveries of all the compounds reported was >94%, excluding oxacarmazapine which was <69% [212]. However, modified POCIS using 600 mg Strata-X resin sorbent and Chemcatcher (SDB-RPS or SDB-XC) at pH 6.5 was successful for the detection of caffeine, carbamazapine, capsone, DEET, hydrochlorothiazide and troclosan [214]. However, Chemcatcher SDB-RPS showed a higher accumulation in comparison with Chemcatcher SDB-XC. Chemcatcher with SDB-RPS, due to having the larger particle size showed less linear accumulation profile however, most neutral pesticides and personal care and pharmaceutical products produce linear accumulation on both Chemcatcher and Strata-X, although, in addition codeine at pH 6.5 showed a linear profile [214]. Furthermore, POCIS was used in the marine environment and 93 pharmaceuticals in 43 different sites were discovered in the Baltic Sea and in the Skagerrac strait, Jutland peninsula of Denmark using Oasis HLB as the POCIS absorbent. In this study, 200 mg HLB sorbent was sandwiched between a polyethersulphone (PES) membrane. Carbamazepine was the most frequently obtained compound present in 37 out of 43 samples and reported to be between 0.57–3.2 ng/L in different locations around the Baltic Sea [215].

**Table 6:** The application of POCIS in the marine environment.

POCIS Resins	Analyte	Year	Country	Elute	Application	Deployment Condition	Adsorbent Quantity	Analyte %Recoveries	Ref.
**Modified POCIS Strata-X and Chemcatcher™** (**SDB-RPS**)	Caffeine, Carbamazapine, Dapsone, DEET, Hydrochlorothiazide, Troclosan	2014	Australia	MeOH, ACN & Ace	Pharmaceuticals	Marine and freshwater Environments (grab sampling & passive sampler)	600 mg PES membranes (47 mm diameter) with 147 mm thickness and a pore size of 0.2 mm (used on Chemcatcher with SDB-RPS) or 140 mm thickness and a pore size of 0.45 mm	Caf: 102% CBZ: 104% Dap: 74% DEET: 77% Hydro: 99% Tro: 84	[214]
**POCIS Oasis HLB**	Carbamazapine (CBZ), Oxacarbamazapine (Ox), and their related metabolites	2014	Mediterranean Sea	MeOH	Pharmaceuticals one POCIS disk was placed into glass aquaria containing 1.5 L of filtered spiked seawater at 5 μg/L	Marine environment (lab experiment)	200 mg	CBZ: 110 ± 4 (5 ng), 95 ± 11 (10 ng) & OX: 58 ± 7 (5 ng), 69 ± 3 (10 ng)	[212]
**POCIS HLB** **PES membrane**	93 pharmaceuticals	2018	Sweden	DCM/ACN (8/2, *v*/*v*), & DCM	Pharmaceuticals	Marine (grab sampling)	200 mg	Conc’s ranging between 0.01 & 80 ng/L	[215]

Abbreviation: MeOH (Methanol), EtOAc (Ethyl acetate), DCM (Dichlorometane), Ace (Acetone), ACN (Acetonitrile).

### 4.2. POCIS and Wastewater Monitoring

The application of POCIS in sampling wastewater treatment discharge to detect compounds such as pharmaceuticals, beta-blockers, pesticides and personal care products over traditional grab sampling methods has been well developed (Table 7, Table 8, Table 9 and Table 10). Six drugs, azithromycin, fluoxetine, omeprazole, levothyroxine were the most frequent prescribed drugs in the United States, and two illicit drugs, methamphetamine and 3,4-Methylenedioxymethamphetamine (MDMA), as polar components were monitored in wastewater effluent using 200 mg Oasis HLB. The recovery values, azithromycin 15–66 ng/L, methamphetamine, 2 ng/L and methylenedioxymethamphetamine 0.5 ng/L showed that although the concentration of these substances in the wastewater is not high there is the concern that accumulation in wastewater can cause chronic effects on human health as well as on native biota, that are continuously exposed to those substances [216].

Oasis HLB is the most popular sorbent used in passive sampling and POCIS in wastewater studies. Studies indicate 200 mg of sorbent is sufficient sorbent mass for sampling analytes from sewage however, there are some other studies that showed 220 mg HLB sorbent to be the optimum mass to sample a range of pharmaceuticals such as neutral pharmaceutical ingredients, antibiotics and illicit drugs [217]. In addition to the amount of sorbent used, other parameters such as sampling rate, temperature and turbulence had an impact on the uptake of these compounds by the sorbent. Furthermore, artificial sweeteners such as sucralose, acesulfame and beta-blockers showed a high affinity to POCIS sorbent. Sucralose (128–213 ng/L) and acesulfame (4–33 ng/L) was reported in treated wastewater [217,218]. In contrast, applying POCIS using 30 mg HLB sorbent for five perfluoroalkyl substances in surface water and hospital wastewater, showed that all five substances could accumulate in POCIS with a concentration range of 6.5 × 10^−3^ to 3.6 × 10^−1^ nmol/L in the influent and accumulate in POCIS at a concentration of 1.3 × 10^−2^–2.2 × 10^−1^ nmol/L in the effluent [136]. Although, the efficiency of HLB in POCIS has been discussed in different studies, the efficiency of some other modified POCIS using different type of sorbents in sampling of biocides, pharmaceutical ingredients, organophosphates, beta-blockers and chlorinated pollutants have also been studied [219]. Octadecyl-functionalized silica gel (C18) and triphasic sorbents were applied in a POCIS device for the extraction and monitoring of alkylphenols (APs), hormones, bisphenol-A (BPA), synthetic musk fragrances and herbicides (trifluralin (Tri) and alachlor (Ala)) in wastewater treatment plant effluent. Determination of the sampling rate and uptake behaviour of those compounds demonstrated that non-polar C18 sorbent shows acceptable results for analysing these compounds which have moderate polarities such as 4t-OP (octylphenol) alkylphenols with 59% recovery, however for other alkylphosphenols compounds with hydrophobic natures such as NPs (nonylphenols) with 47% recovery, 4n-OP alkylphenols with 38% recovery and MeEE2 with 15% recovery, non-polar C18 is not a satisfactory sorbent [219].

POCIS is also known and accepted as a powerful and reliable tool in monitoring a wide range of non-pharmaceuticals, Challis et al. assessed the application of POCIS in the evaluation of non-targeted analytes for high resolution mass spectrometry of wastewater. They presented this study in comparison with the application of organic-diffusive gradients in thin-films (o-DGT) for the determination of both targeted and non-targeted compounds. The efficiency of using 200 mg Oasis HLB in POCIS for the evaluation of neonicotinoids, organophosphates, triazines, antibiotics, b-blockers, SSRI’s and sodium channel blockers was compared to o-DGT samplers which were constructed using two layered gels made of 1.5% agarose. This study suggested o-DGT was more reliable for TWA (Time-Weighted Average) determination contaminant concentrations during the deployment period, this is due to the o-DGT feature, having a diffusive hydrogel membrane consisting of 96–98% water together with the control of the viscosity of water, which reduces the water boundary layer effects [201].

**Table 7:** Application of POCIS on wastewater.

POCIS Resins	Analyte	Year	Country	Elute	Application	Deployment Condition	Adsorbent Quantity	Analyte %Recoveries	Ref
**Oasis HLB**	Omeprazole, fluoxetine, azithromycin, levothyroxine, methamphetamine, and methylenedioxymethamphetamine	2004	USA	MeOH	Pharmaceuticals	WW River water Deployed	200 mg	Azithromycin 15–66 ng/L, methamphetamine, 2 ng/L, methylenedioxymethamphetamine 0.5 ng/L	[216]
**POCIS Oasis HLB**	25 pharmaceuticals and personal care products	2007	Canada	MeOH	Pharmaceuticals Uptake rates were 0.040 to 2.462 L/d in uptake rates between 0.016 and 0.223 L/d	WW and SW	200 mg	RS values for 13 of the 25 analytes could be determined	[220]
**Oasis HLB** **PES membrane**	A range of Pharmaceuticals, personal care products, endocrine disrupting	2010	Switzerland	MeOH	Pharmaceuticals, personal care products, endocrine disrupting	Treated WW Flow rate 2.6 and 37 cm/s	200 mg	Different recoveries based on flow rate is reported	[221]
**Oasis HLB (pharmaceutical & pesticide), PES membrane**	A range of pharmaceuticals, hormones and pesticide are reported	2011	US	MeOH	Pharmaceuticals, steroid hormones, pesticides	WW	200 mg	Recoveries as reported in the paper	[222]
**Oasis HLB** **PES membrane**	Beta-blockers and hormones	2012	France	MeOH containing 5% NH_4_OH.	Pharmaceuticals, hormones	WW	200 mg	Hormones low conc’s prevented determination of reliable sampling rates. Suitable for b-blockers	[223]
**POCIS Oasis HLB**	Atenolol, Prednisolone, Methylprednisolone, Sulfamethoxazole, Ofloxacin, Ketoprofen	2013	France	MeOH	Pharmaceuticals Uptake profiles (25 °C, flow velocity 0.16 m/s), an automatic sampler, taking 100 mL every 15 min between 7:00 a.m. and 9:00 p.m. and 100 mL every 30 min during the night.	Hospital sewage	200 mg	64–95%	[224]
**SBSE** **Oasis HLB**	19 moderately hydrophobic to hydrophobic pesticides	2013	France	MeOH then, MeOH/EtOAc (75:25, *v*/*v*)	Pesticide	Agricultural WW	200 mg	SBSE was able to integrate a concentration peak triggered by a quick flood	[225]
**POCIS HLB** **PES membrane**	Naproxen, Ibuprofen, triclosan	2014	South Africa	ACN, then ACN: MeOH, 50:50 (*v*/*v*),	Pharmaceuticals	WW	200 mg	Naproxen 92%, Ibuprofen 108%, triclosan 75%	[226]
**POCIS HLB**	Acetaminophen, caffeine, 1,7-dimethylxanthine, cotinine, dextroamphetamine, diethyltoluamide (DEET), diphenhydramine, ibuprofen, methamphetamine, carbamazepine, azithromycin, erthyromycin, lincomycin, monensin, sulfachloropyridazine, sulfamethazine, sulfadimethoxine, sulfamethazole, sulfamethoxazole, sulfamerazine, sulfathiazole, thiabendazole, tiamulin, tylosin, and ractopamine	2015	Nebraska	MeOH	Pharmaceuticals	WW effluents	200 mg	Results available for two different locations	[227]
**POCIS HLB, PES membrane**	17-β-estradiol, 17-α-estradiol, 17-αethinylestradiol, estrone and estriol	2016	Czech republic	DCM, Ace, MeOH	Hormones	WW	200 mg	3.4 8 to 3.66 at nominal steroid concentration in water 100 ng/L	[228]
**POCIS HLB**	A range of Pharmaceuticals, artificial sweeteners, food additives, antibiotics personal care product, fragrances, sugar substitutes, steroid hormone	2016	Canada	MeOH	Pharmaceuticals, artificial sweeteners, food additives, antibiotics personal care product, fragrances, sugar substitutes, steroid hormone	WW	220 mg	Detected concentrations discussed	[217]
**POCIS HLB**	Ciprofloxacin	2016	France	ACN	Pharmaceutical	Hospital effluents	200 mg	Indicate a potential ecotoxicological risk	[229]
**POCIS HLB** **PES membrane**	Atrazine, carbendazim, desethylatrazine, desethylterbutylazine, diuron, S-metolachlor, terbutylazine, alprazolam, atenolol, carbamazepine, diazepam, diclofenac, ibuprofen, naproxen, 17-alpha-estradiol, 17-alpha-ethinylestradiol, 17-beta-estradiol, estriol, estrone, (BDE 28, BDE 47, BDE 99, BDE 100, BDE 153, BDE 154, PFOA, PFOS, Bisphenol A, triclosan	2016	Germany	-	Pesticides, pharmaceuticals, hormones, fluorinated surfactants, bisphenol A, triclosan	Treated WW	200 mg	Recovery details discussed	[204]
**C18 & triphasic**	Alkylphenols (APs), several hormones, bisphenol-A (BPA), synthetic musk fragrances and herbicides, e.g., trifluralin (Tri) & alachlor (Ala), DES hormones	2016	Germany	DCM/EtOAc/MeOH (4:4:2, *v*/*v*)	Herbicides, alkyphenols, hormones	WW treatment plant	200 mg	Recovery percentages vary between 6% for DES to 96% for Tri	[219]
**POCIS OASIS HLB**	A range of Pharmaceuticals and illicit drugs	2017	Norway	5% NH_4_OH in MeOH, and 5% HOAc in MeOH	Pharmaceuticals and illicit drugs	WW	220 mg	Results discussed in the paper	[218]
**POCIS HLB** **PES membrane**	PFHxA, PFOA, PFHxS, PFDoDA, PFOS	2017	China	MeOH containing 5% NH_4_OH	Perfluorinated compounds	WW	30 mg	Concentration shown on diagram	[136]
**POCIS HLB**	12 pharmaceuticals	2017	Ukraine	MeOH	Pharmaceuticals	Surface water Hospital WW	200 mg	Removal patterns of pharmaceuticals were discussed based on specific physical chemical properties of molecules	[230]
**POCIS HLB** **MIP membrane**	BTEX, chlorinated pollutants and pharmaceuticals	2017	Czech republic	MeOH, & MeOH/DCM (1:1, *v*/*v*), then MeOH	Pharmaceuticals	Water remediation	200 mg	(POCIS) for the pharmaceuticals and in situ soil microcosms for microbial community analysis, was proven	[231]
**POCIS HLB** **PES membrane**	Carbamazepine (CBZ) and sucralose (SCR)	2018	Brazil	MeOH/water (1:2, *v*/*v*),	Pharmaceuticals	Sewage	200 mg	CBZ: <LOD 3.6 ng/g, SCR: <LOD 139.9 ng/g	[232]
**POCIS**	Clarithromycin, metoprolol, propranolol, carbamazepine, sulfamethoxazole, Atenolol	2018	Canada	-	Pharmaceuticals	WW	-	Recoveries compared over three years	[233]
**Modified- POCIS Strata-X PES membrane**	8 organophosphate flame retardants (OPFRs)	2018	China	MeOH	Pharmaceuticals and their metabolites	WW	200 mg	Results discussed	[234]
**Oasis HLB PES membrane**	Biocides, carbamazepine, diclofenac, terbutryne, diuron, carbendazime	2020	Luxembourg	DCM/ACN (1:1, *v*/*v*),	Pesticides, pharmaceuticals	WW	200 mg	Results discussed in different flooding time	[203]
**ODGT and POCIS Oasis HLB PES membrane**	Neonicotinoids, organophosphates, triazines, antibiotics, b-blockers, SSRI’s, and sodium channel blockers	2020	Canada	-	Pharmaceuticals	WW	200 mg	Quantitative comparison of o-DGT, POCIS is discussed	[201]

Abbreviation: MeOH (Methanol), EtOAc (Ethyl acetate), DCM (Dichlorometane), Ace (Acetone), ACN (Acetonitrile), HOAc (Acetic acid), NH_4_OH (Ammonium hydroxide), Wastewater (WW), Surface water (SW).

### 4.3. Application of POCIS to the Detection of Pollutants in Freshwater, Rivers, Lakes and Drinking Water Sources

Residues of personal care products including cosmetics, UV-filters, pesticides, pharmaceuticals including beta-blockers, herbicides, and pesticides in freshwater lakes and rivers is a matter of public concern and monitoring of lakes and reservoirs is necessary to evaluate the amount of pharmaceutical and personal care products (PPCPs) in drinking water pre and post treatment [235,236].

A range of pharmaceutical components from different biological classification systems (BCS) [237] and household materials along with personal care products, were monitored and identified as present in several studies [220,238]. These compounds can be introduced to surface and ground water through different routes such as household waste, clinical wastewater and landfill leachate [235].

Pharm-POCIS has been used to sample a range of pharmaceuticals, pesticides, microcystins, plasticisers and UV filters used in cosmetic personal care products from wastewater, groundwater, river and lakes [207].

Hydrophilic-Lipophilic Balanced sorbent (Oasis HLB) and Triphasic sorbent mixture [207] are commonly used sorbent phases, Oasis HLB dominates studies as the sorbent resin with the highest efficiency, although the triphasic admixture, Strata-X, Strata-XW, also demonstrate reliable results. Oasis HLB can be used to detect compounds with hydrophilic tendencies (such as benzene) and those exhibiting hydrophobic behaviour, e.g., aliphatic chains and pyrrolidone, and is suitable for the separation of low molecular weight and polar components. However, water samples containing high polarity compounds such as atrazine, desisopropylatrazine (DIA), diuron and dicamba-d_3_ with an acidity level of pKa < 4.5 reach thermodynamic equilibrium level with the HLB membrane quickly, thus preventing and reducing accumulation on the sorbent and limiting its use for quantitative studies [239]. However, an investigation that involved increasing the amount of sorbent from 200 mg to 600 mg showed a higher efficiency in the accumulation of highly acidic (bentazon, dicamba, mesotrione, and metsulfuron) and polar (atrazine and diuron) compounds from water samples [239]. In contrast, there are other studies that show a lower concentration of Oasis HLB sorbent such as 2.75, 5.55 and 11.10 mg/cm^2^ having lower mass to surface area ratio which results in a higher efficiency by creating a thin layer of sorbent that increases the water speed into the sorbent and thus increased accumulation of compounds [168]. In addition, Muller demonstrated the bioavailability of endocrine disrupting chemicals (EDS) from suspended sediments (that occurs due to flood events) on HLB sorbent. In the bioavailability screening of estrogenic compounds using 54.5 ± 0.5 mg Oasis HLB, this phase showed reliable analytical results for the following list of compounds (nonylphenol detected at 18 mg/L concentration, estrone (E1) detected at 14 ng/L concentration, 17β-estradiol (E2) detected at 0.2 ng/L concentration and 17β-ethinylestradiol EE2 detected at 0.5 ng/L concentration) [240].

An application of POCIS using 200 mg Oasis HLB for in situ studies on the most frequent prescribed drugs examined the efficiency of POCIS for monitoring azithromycin, fluoxetine, levothyroxine, omeprazole and sulfamethazole and recorded 100%, 95%, 86%, 95% and ~100% recoveries, respectively [206,241,242,243].

Other pharmaceutical recovery studies report reasonable recovery rates for carbamazepine, caffeine, aspirin, diazepam, naproxen, theophylline, amitriptyline at 71%, 77%, 66%, 105%, 61%, 81% and 97%, respectively. Other compounds detected reported recovery vales of 103%, 99%, 94%, 98% for alph-ethynylestradiol, beta-estradiol, levonorgestrel and progestron, respectively. In addition, in the same study the recovery ratios of pesticides were measured and included the following compounds diazinon, diuron, dimethacholor, atrazine, CDPU, CDPMU with 117%, 94%, 148%, 87%, 73% and 71% recoveries, respectively [244]. A separate study on 21 pharmaceuticals using 200 mg Oasis HLB accumulated from river water showed the highest recovery values for alprazolam, clenbuterol, bromazepam, ibuprofen and theophylline with 97%, 92%, 93%, 90% and 90% recoveries, respectively. In addition, the lowest recovery value reported was for aspirin at 51% [245]. Estragon steroid hormones were also evaluated using POCIS apparatus with 230 mg Oasis HLB used for monitoring oestrogens, oestrone (E1) and 17β-oestradiol (E2), 17α-ethynylestradiol (EE2) and diclofenac. The limit of detection (LOD) reported was 0.001 mg/L for pharmaceutical and endocrine disrupting chemicals (EDC) [246].

Eight classes of pesticides, carbamates, chloroacetanilides, dicarboximides, morpholines, organophosphorous, phenylureas, strobilurines and triazines were studied using POCIS and three different amounts of sorbent (60 mg, 150 mg, 500 mg) by Lissalde et al. [16]. Reasonable recoveries of 75% was reported for the test analytes using 60 mg of sorbent however, the recoveries using 150 mg and 500 mg of sorbent were much lower, reported at 22.8% and 33.6%, respectively. The examination of polar pesticides in ground water in France, using a pharma-POCIS with 450 mg Oasis HLB resin showed the enhanced ability of applying passive samplers over traditional manual sampling methods in detecting trace substances in ground water [247].

Other resin sorbents studies for detecting pharmaceutical and pesticides in river and ground water, have used Strata-X using a nylon Membrane and mixed Polymer Sampler (MPS) [248,249,250,251]. A copolymer of poly(divinylbenzene)-N-vinylpyrrolidone was used as resin sorbent for monitoring 46 pesticides, 17 pharmaceuticals and some artificial sweeteners [252]. Strata-X CW was chosen for monitoring pollutants such as pharmaceuticals, pesticides and corrosion inhibitors in river water, and this resin sorbent was compared with Oasis HLB [253]. The recovery results for Strata-X CW and Oasis HLB were 18–75% and 64–97%, respectively, which shows that although the same amount of sorbent, 200 mg, was used in both studies, the hydrophilic-lipophilic balance sorbent (Oasis HLB) had better results than Strata-X in the up-take of compounds. However, in this study metformin was an exceptional compound showing only 1% recovery [253]. In addition, a modified POCIS device using 600 mg Strata-XAW and a polyrthersulfone (PES) membrane to assess the effects of water velocity on the concentration of the analyte, was used for monitoring prefluorinated chemicals (PFCs) in river water. A comparison between PFCs with a molecular weight ≤ 464 and PFCs with molecular weight ≥ 500 showed that by increasing water flow rate, the sampling rate improved for those PFCs with molecular weight ≤ 464 [254].

Several lake water pollution studies demonstrated the capability of POCIS with Oasis HLB to detect pesticides and herbicides. A 200 mg device was used to evaluate pesticides such as neonicotinoid insecticides (NNIs), thiamethoxam, clothianidin, imidacloprid, [255] atrazine, azadirachtin, carbofuran, chlorpyrifos, cypermethrin, dieldrin, imidacloprid, and profenofos (0.1 μg/L at 30.8 ± 1.3 °C) [256]. Additionally, atrazine, diurom, 2,4-D, mecoprope, fluconazole, climbazole and chlorothalonil were evaluated in the western lake of Ontario [257]. The concentrations of endocrine disruptions such as bisphenol A, 17β-estradiol, estrone and 4-nonylphenol in fish in lakes were detected in ppm concentration levels [258]. In addition, monitoring pharmaceuticals in lake water has been reported for 35 active pharmaceutical ingredients (APIs) with the recoveries of 26 compounds detected between 80–120% in which it appeared that 60 mg HLB is appropriate for the evaluation of 1 L sample quantities [257,259]. Brophy et al. assessed microcystin-LR (MC-LR) absorption using POCIS with 220 mg Oasis HLB resin in lake water, the sampling rate showed 0.045 and 0.041 L/day for concentrations of 0.5 and 1 µg/L, respectively. These values detected from POCIS are higher than the MC-LR concentrations detected from the traditional grab sampling method. The grab sampling method had a value of 0.3 µg/L MC-LR for its detection limit and for the POCIS the detection limit was 1 ng/L, per day [260].

Studies on drinking water confirm Oasis HLB to be the most used sorbent for monitoring clothianidin at 300 µg/L concentration, imidaclopid at 500 µg/L concentration and thiamethoxam at 5 µg/L concentration with POCIS [261,262]. Oasis HLB and DOWEX sorbents were compared for the sampling of pesticides in drinking water. Recovery averages for Dowex Optipore L-493 was 90% (range between 66–127%), and for HLB was 91% (range between 66–135%) [263,264]. A modified POCIS sampler using two different sorbents, Chromabond HR-X and Oasis MAX were compared for monitoring acidic herbicides in drinking water. This comparison showed that Oasis MAX had >85% elute recovery, a higher sampling rate compared with HR-X which had <20% recovery [265]. Further studies reported the detection of pharmaceuticals, alkylphenols, hormones, UV filters [266] personal care products [263,264] endocrine disrupting and drugs of abuse [262] and prefluorinated compounds [267] in drinking water. Microcystin levels in drinking water reservoir during two vegetation seasons were determined, using 90 mg Oasis HLB. The results after 14 days deployment showed a concentration of 1–12 ng/L for the toxin, this detected concentration level was not a risk to human health [268].

**Table 8:** Application of POCIS to river water studies.

POCIS Resins	Analyte	Year	Country	Elute	Application	Deployment Condition	Adsorbent Quantity	Analyte %Recoveries	Ref
**Oasis HLB**	Azithromycin; Fluoxetine; Levothyroxine; Omeprazole	2004	UK	MeOH	Pharmaceuticals	River, deployment at 8 sites	200 mg	Ranging between 86–100%	[241]
**Oasis Tribasic admixture**	Atrazine, Diazinon, Diuron, 17a-Ethynylestradiol, Isoproturon	2004	UK	MeOH	Pharmaceuticals	River, deployment at 8 sites	200 mg	Ranging between 88–99%	[241]
**Oasis HLB** **PES, PE membrane**	Estrone, 17-estradiol, 17-ethynylestradiol, bisphenol A, propranolol, sulfamethoxazole, meberverine, thioridazine, carbamazepine, tamoxifen, indomethacine, diclofenac and meclofenamic acid in sewage effluent and river water.	2008	UK	MeOH	Pharmaceuticals, endocrine disrupting compounds, personal care products	River	100 mg	Propranolol, sulfamethoxazole, carbamazepine, indomethacine & diclofenac, varied between 3.0 & 45.6 ng L^−1^, <LOD & 17.6 ng L^−1^, 16.6 and 539 ng L^−1^, 0.4 & 7.2 ng L^−1^ & 2.4 & 65.2 ng L^−1^, respectively; applying POCIS, conc’s were between 2.8 & 40.5 ng L^−1^, <LOD &18.2 ng L^−1^, 26.3 & 427 ng L^−1^, 0.5 & 11.9 ng L^−1^ & 4.4 & 165 ng L^−1^, respectively.	[242]
**Oasis HLB, PES membrane**	Diuron, (1-(3,4-dichlorophenyl) urea (DCPU), 1-(3,4-dichlorophenyl)-3-methylurea (DCPMU)	2010	France	MeOH, & 75% MeOH/25%, EtOAc (*v*/*v*),	Herbicides	River	500 mg	Conc’s of diuron and its transformation products in microcosm	[269]
**Oasis HLB, PES membrane**	20 pesticide analytes	2010	USA	MeOH	Pesticides, polycyclic, aromatic hydrocarbons	Ground water cave	200 mg	Vary during different month	[270]
**Strata-X, PES membrane**	E1, E2, EE2	2010	UK	Ethyl acetate, & MeOH & ultrapure water	Endocrine disrupting substances	River	300 mg	Ranging between 0.9–2.2 ng/L	[250]
**Strata-X, PES membrane**	Prometryn	2011	Germany	MeOH	Prometryn	River	300 mg	0.01–0.07 mg/L	[251]
**Oasis HLB, PES membrane**	Desethylatrazine, Deisopropylatrazine, Simazine, Desethylterbuthylazine, Atrazine, Metolachlor, Terbuthylazine	2011	France	MeOH, 75% & MeOH/25% ethyl acetate (*v*/*v*)	Pesticide	River deployment	200 mg	After 24 h terbuthylazine > atrazine > simazine > metolachlor. Daily conc. varies	[10]
**Oasis HLB, PES membrane**	Range of substances in different pH reported	2011	Canada	MeOH	Pharmaceuticals, personal care products, disrupting substances	River & Tap water (Water chamber in lab)	200 mg	Recoveries in different pH are reported	[174]
**Oasis HLB, PES membrane**	A wide range of pollutants Pharmaceuticals, Polycyclic aromatic hydrocarbons, Hormones, Pesticides, Phenols	2011	France	DCM/MeOH (50:50 *v*/*v*),	Pharmaceuticals, polycyclic aromatic hydrocarbons, hormones, phenols	River deployment	200 mg	Recovery tables are reported in the paper	[244]
**Oasis HLB**	33 Pesticides	2011	France	MeOH & MeOH/ethyl acetate, 75/25 (*v*/*v*)	Pesticide	River	60 mg, 150 mg, 500 mg	Dimetomorph: 14.8 ng/L, linuron: 5.1 ng/L, metazachlor: 11.3 ng/L, terbuthylazine: 4.8 ng/L	[16]
**Oasis HLB, PES membrane**	Alkylphenols, Phenolated polymer, Oestrogenic hormones, Antidepressants, Anti-inflammatory, b-Blockers, Hypolipidemic agent	2012	France	-	Alkylphenols, phenolated polymers, hormones, pharmaceuticals	River and wastewater treatment plant	200 mg	Results shows the diagnostic capacity of POCIS tools	[271]
**Oasis HLB, PES membrane**	Sulfamethoxazole	2012	Czech Republic	MeOH:water (9:1 *v/v* acidified with 0.1% TFA)	Pharmaceuticals Sulphonamides in stream	River	200 mg	20 up to 736 ng/L	[243]
**Oasis HLB, PES membrane**	Perfluorinated alkylcarboxylates	2012	Australia	0.1% (*v*/*v*) ammonia solution in MeOH, then MeOH	Perfluorinated chemicals	Harbour	200 mg	0.1−12 ng/L	[272]
**Oasis HLB, PES membrane**	Range of pharmaceuticals	2012	France	MeOH, MeOH/DCM mixture (50:50), & DCM	Pharmaceuticals	River	200 mg	Ranging between 51–97%	[245]
**Oasis HLB** **PES membrane**	A range of 14 different pesticides	2012	France	-	Pesticide	River	200 mg	Concs. discussed	[206]
**Oasis HLB** **PES membrane**	Chloro, Propic a, Propic b, Hex, Phos	2012	USA	MeOH	Pesticide	Synthesized river water (Effect of flow velocity was assessed)	200 mg	Levels of organic carbon (<0.1–5 mg/L)	[273]
**Oasis HLB** **PES membrane**	21 pharmaceuticals, 6 alkylphenols and 27 hydrophilic pesticides and biocides	2012	France	MeOH & MeOH/DCM (*v*/*v*: 50/50), & DCM	Pharmaceuticals, alkylphenols and pesticides	Surface water	200 mg	Ranging between 2.5–33 ng/L	[274]
**Pharma-POCIS Oasis HLB**	Polar pesticides	2013	France	MeOH	Pesticide	Ground water Deployed in 15 m depth and drinking water	450 mg	POCIS could be tested on groundwater sites which present temporal variations in concentrations for studying its integrative capacity	[247]
**Strata XAW, PES membrane**	Range of prefluorinated chemicals	2013	Australia	0.1% (*v*/*v*) ammonia sol in MeOH & MeOH	Perfluorinated chemicals	River	600 mg	Ranging between 71–92%	[254]
**POCIS HLB** **PES membrane**	Terbuthylazine, diuron and linuron	2014	Switzerland	MeOH	Herbicides	River	200 mg	Terbuthylazine: 220 ng/L, diuron: 70 ng/L linuron: 50 ng/L	[275]
**POCIS HLB**	Range of pesticides	2014	France	MeOH	Pesticides	River	200 mg	Ranging between 138–1080 ng/L	[276]
**POCIS HLB, PES membrane**	23 polar pesticides and 8 metabolites	2014	France	MeOH, & MeOH/ethyl acetate, 75:25 (*v*/*v*)	Pesticides	River	200 mg	Details discussed in the paper	[277]
**POCIS HLB, PES membrane**	Atrazine	2014	Canada	MeOH	Pesticides	River	200 mg	Atrazine conc. in 24 streams discussed	[278]
**POCIS HLB** **PES membrane**	Atrazine, propazine, terbutylazine, diclofenac, ibuprofen, ketoprofen, perfluorooctanoic acid and perfluorooctanesulfonate	2014	Italy	Acetone	Perfluorinated chemicals, pharmaceuticals, pesticides	River (linear velocity of 2.0, 5.1, 10.2 and 15.3 cm/s)	200 mg	Spiked conc. in different flow velocities is shown	[279]
**POCIS HLB** **PES membrane**	Diuron	2014	France	MeOH, & MeOH/DCM (*v*/*v*: 50/50), then DCM	Pesticides	Coastal water	200 mg	Oysters were exposed to diuron integrated conc’s as low as 0.2 and 0.3 g/L	[280]
**POCIS HLB**	39 pesticides and metabolites	2015	France	MeOH, & MeOH: EtOAc, 75:25 (*v*/*v*)	Pesticides and metabolites	River	200 mg	Frequency of detection and concentration reported	[281]
**POCIS HLB**	12 veterinary antibiotics	2015	USA	MeOH	Pharmaceuticals	River	200 mg	Conc’s ranging from 0.0003 ng/L to 68 ng/L	[282]
**POCIS HLB, PES membrane**	APIs and pesticides	2015	Portugal	MeOH & DCM/MeOH (50:50; *v*/*v*), & DCM	Pharmaceuticals, pesticides	River	200 mg	Caffeine: 804 ± 209 ng/L, theophylline:184 ± 44 ng/L, Carbendazim: 45 ± 18 ng/L, atrazine, diuron, Isoproturon and simazine levels were below the Environmental Quality Stds	[283]
**POCIS-Pest and POCISPharm, PES membrane**	Organ halogen herbicides, organophosphorous pesticides, carbamate, triazine, urea, pharmaceuticals, phenols, and industrial chemicals	2016	Greece	Hexane/DCM Additionally, DCM/EtOAc (50:50 *v*/*v*)	Pesticides, carbamate, triazine, urea, pharmaceuticals, phenols, and industrial chemicals	River	200 mg	Most compounds showed recoveries ranged from 60 to 110%. The coefficient of variation (CV) ranged from 0.84 to 23.8%. LOD and LOQ ranged from 6.4 to 40.1 ng/L and from 21.5 to 134 ng/L, respectively.	[284]
**POCIS sorbents, HLB and Strata X-CW, PES membrane**	Benzotriazole, methylbenzotriazole, atrazine, diuron, isoproturon, linuron, metolachlor, penconazole, terbuthylazine, carbamazepine, diclofenac, metformin, sulfamethoxazole	2016	Switzerland	MeOH	Corrosion inhibitors, pesticides, pharmaceuticals	River	200 mg	Rs: Benzotriazole: 0.134, methylbenzotriazole: 0.148, atrazine: 0.26, diuron: 0.15, isoproturon: 0.254, metolachlor: 0.139, terbuthylazine: 0.197, carbamazepine: 0.231, diclofenac: 0.165, sulfamethoxazole: 0.103	[253]
**POCIS HLB** **PES membrane**	Metaldehyde, isoproturon, simazine, chlorotoluron, atrazine, epoxiconazole, chlorpyrifos, cypermethrin and permethrin	2016	UK	Ethyl acetate	Pesticides	River	200 mg	Results compared in three different sites	[285]
**POCIS HLB**	In vitro (i.e., zf liver cell lines stably expressing zfERα, zfERβ1 and zfERβ2 subtypes) and in vivo (i.e., transgenic cyp19a1b-GFP zf embryos)	2016	France	MeOH, & DCM/MeOH (50:50; *v*/*v*), & DCM	Endocrine disrupting substances	River	200 mg	Results in different sites discussed	[202]
**POCIS HLB** **PES membrane**	20 parent compounds (PCs) and 11 characteristic TPs in four 11 wastewater-impacted rivers	2016	Sweden	-	Pharmaceuticals	River	200 mg	Results of four different rivers discussed	[192]
**POCIS HLB** **PES membrane**	CBZ: carbamazepine, CAF: caffeine, BPA: bisphenol A, LIN: lincomycin, SFA: sulfamethazine, SFO: Sulfamethoxazole, ATZ: atrazine, GEM: gemfibrozil	2016	Singapore	-	Pharmaceutical	River	200 mg	Sediment concentrations for carbamazepine (r = 0.79, p b 0.001), caffeine (r = 0.93, p b 0.001) but not BPA (p = 0.16)	[286]
**POCIS HLB** **PES membrane**	Complex mixtures of micropollutants, including emerging substances or transformation products	2016	France	ACN, MeOH	Rodenticide, hormones, antiparasitic, cardiovascular agent, pharmaceuticals, pesticides and their metabolites	Groundwater	200 mg	Results of two different sites discussed	[287]
**POCIS HLB** **PES membrane**	Tebuconazole, Propiconazole, Carbendazim, Azoxystrobin, Myclobutanil, Iprodione Fluconazole, Ketoconazole, Climbazole, Mecoprop, Agriculture, Dicamba, 2,4-D, Irgarol 1051, Terbutryn, Estrone Natural estrogen, Androstenedione, Ibuprofen, Acetaminophen, Naproxen, Trimethoprim, Sulfamethoxazole, Gemfibrozil, Carbamazepine, Sucralose	2016	Canada	MeOH	Pharmaceuticals, steroid hormones, the artificial sweetener, sucralose, fungicides, herbicides, biocides	River	200 mg	Results compared in different sites	[288]
**Passive sampler copolymer of poly(divinylbenzene)-N-vinylpyrrolidone**	46 pesticides, 17 pharmaceuticals, 1 stimulant (caffeine) and 1 artificial sweetener (sucralose)	2017	France	MeOH, & MeOH/EtOAc (50/50 *v*/*v*) and EtOAc	Pharmaceuticals Average flow of river over ten years 1.0 m^3^/s	The Marque River because of agricultural activities	200 mg	Atrazine 0.22 L/day, Cyprodinil 0.22 L/day, Desethylatrazine 0.09 L/day, Desisopropylatrazine 0.09 L/day, Diclofenac 0.08 L/day, Dimethenamid 0.20 L/day, Isoproturon 0.16 L/day, Metolachlor 0.17 L/day, Metalaxyl 0.19 L/day	[252]
**POCIS HLB, PES membrane**	13 parent pharmaceuticals and 8 of their transformation products (TPs)	2017	China	MeOH	Pharmaceuticals and their metabolites	River	200 mg	The max concentration: 544.0 ng/L (CBZE), and the minimum value was 0.43 ng/L1 (SDZ)	[289]
**POCIS HLB, PES membrane**	45 pesticides	2017	France	MeOH	Pesticides	Surface water in vitro	200 mg	Average concentrations discussed in the paper	[290]
**POCIS HLB**	A range of pesticide, fungicide, herbicide, and insecticide	2017	USA	MeOH	pesticides	Surface water	200 mg	A total of 141 compounds detected at one or more of the 97 sites sampled	[291]
**POCIS HLB, PES membrane**	37 pharmaceuticals and 3 human tracers	2018	France	MeOH, & MeOH/EtO Ac, 75:25 *v*/*v*	Pesticides and their Metabolites, pharmaceuticals	River	200 mg	Frequency and concentrations in the paper	[292]
**POCIS**	Organophosphate flame retardants (OPFRs)	2018	China	Ethyl acetate	Endocrine disrupting substances	River	200 mg	Six sampling locations ranged from 8.99 to 112.45 ng/L with an average concentration of 47.04 ng/L	[293]
**POCIS HLB, PES membrane**	17 pharmaceuticals, pesticides, per- and polyfluoroalkyl substances (PFASs)	2018	USA	0.1% (*v*/*v*) ammonia solution in MeOH, & MeOH	Pesticides, pharmaceuticals, and perfluorinated chemicals	River	200 mg	Concentration shown during different month of the year	[294]
**POCIS HLB, PES membrane**	Atrazine, thiamethoxam, clothianidin, imidacloprid, 2,4-D and carbamazepine	2018	Canada	MeOH	Pesticides and pharmaceuticals	River	200 mg	Recoveries compared during years	[295]
**POCIS Oasis HLB**	37 pharmaceuticals and 3 human traces	2018	France	75:25 *v*:*v* MeOH:Ethyl	Pharmaceuticals flow rate at each sampling points was calculated proportionally at the size of sampling point watershed (75, 145 and 55 km^2^)	agricultural rural headwater river	200 mg	23 compound out of 37 detected	[238]
**POCIS HLB, PES membrane & Mixed Polymer Sampler (MPS)**	Alachlor, atrazine, cybutryne, diclofenac, diuron, isoproturon, PCP, Simazine, terbutryne	2018	Germany	MeOH	Pharmaceuticals, pesticides	River	220 mg	Dissolved concentration of the compounds shown	[249]
**POCIS**	S-metolachlor	2018	France	-	Pesticides	River	200 mg	Concentration discussed in different sites	[296]
**POCIS HLB, PES membrane**	Malathion, diuron, carbofuran, carbendazim, trifluralin, imidacloprid, metolachlor, and acetamiprid	2018	Brazil	MeOH	Pesticides	River	220 mg	Malathion 7.7%, diuron 5.1%, carbofuran 35.9%, Carbendazim 12.8%, trifluralin 5.1%, imidacloprid 5.1%, metolachlor 7.7%, acetamiprid 2.6%	[297]
**POCIS HLB, PES membrane**	Atrazine, 2,6-dichlorobenzamide, bentazone, chloridazon, isoproturon, and propiconazole	2018	Sweden	Ethyl acetate	pesticides	Surface water	220 mg	Herbicides 36%–48%, fungicides 36%–21%, metabolites 11%–12%, insecticides 8%–10%, and other or mixed types 8%–10%	[298]
**POCIS HLB**	32 selected herbicides, fungicides, and insecticides (mainly polar)	2018	Germany	ACN	pesticide	Surface water	230 mg	Details discussed in the paper	[299]
**POCIS HLB, PES membrane**	A range of pesticides	2018	Japan	MeOH	Pesticides	River	220 mg	Compared POCIS with grab sampling	[300]
**Strata XAW & HLB** **Nylon membrane**	Acetaminophen, atrazine, diuron and norfloxacin hydrochloride, amitriptyline, irbesartan, ketoprofen and progesterone	2018	Spain	2.5% methanolic ammonia & MeOH	Pesticide, pharmaceuticals	Estuarine	100 mg each	Feasibility of the simultaneous uptake of hydrophilic, acidic and basic compound	[248]
**Oasis HLB, PES membrane**	Estrone (E1), Nonylphenol (NP), 17b-estradiol (E2), ethynylestradiol (EE2),	2019	Germany	-	Endocrine disrupting substances	River water	54.5 mg	NP 18 mg/L, E1 14 ng/L, E2 0.2 ng/L, EE2 0.5 ng/L	[240]
**Oasis HLB**	20 pesticides and 32 point source chemicals	2019	Spain	MeOH	Pharmaceuticals, pesticides, hormones	River	200 mg	Recovery % is reported in the paper	[301]
**POCIS**	20 pesticides, and 32 point source chemicals, mainly pharmaceuticals	2019	Spain	_	Pharmaceuticals, pesticides, hormones	River	-	High recoveries reported	[302]
**Oasis HLB, PES membrane**	Biomarkers of estrogenic endocrine disruption in smallmouth bass	2019	USA	DCM/MTBE 80:20 (*v*/*v*),	Endocrine disrupting substances	River	200 mg	Ranging between 28–92%	[303]
**Oasis HLB, PES membrane**	Pharmaceuticals, endocrine disrupting substances, pesticides	2019	Ireland	MeOH	Pharmaceuticals, endocrine disrupting substances, pesticides	River	230 mg	Conc’s in different years reported	[246]
**Oasis HLB, PES membrane**	168 targeted compounds	2019	Slovakia	MeOH/DCM, (1:1 *v*/*v*)	Pesticides, pharmaceuticals, hormones, polycyclic aromatic hydrocarbons, polychlorinated biphenyls	River	200 mg	Risk assessment of the detected compounds revealed	[304]
**Oasis HLB** **PES membrane**	A range of pesticide, herbicide, fungicide, metabolite, and insecticide	2019	France	MeOH, & MeOH/ethyl acetate, 75:25 *v*/*v*	pesticides	River	200 mg Bags deployed based on the depth >100 m or <100 m	Results discussed in the paper	[305]
**Oasis HLB** **Six different membrane**	25 pharmaceuticals and personal care products	2022	USA	Formic acid: MeOH 88:12%	Pharmaceuticals and personal care products	Vernal pools		Results compared to grab sampling in the paper	[306]

Abbreviations: MeOH (Methanol), EtOAc (Ethyl acetate), Hx (Hexane), Rs = Sampling Rate.

**Table 9:** Application of POCIS on Lake water.

POCIS Resins	Analyte	Year of Study	Country	Elute	Application	Deployment Condition	Adsorbent Quantity	Analyte %Recoveries	Ref
**Oasis HLB** **PES memberane**	Range of substances reported	2010	USA	MeOH	Endocrine disrupting	Lake	200 mg	Recoveries reported	[258]
**Oasis HLB** **PES membrane**	Ibuprofen, Gemfibrozil, Caffeine, Carbamazepine, Trimethoprim, Venlafaxine, desmethyl-venl, Citalopram, Galaxolide, Tonalide, Triclosan	2012	Canada	MeOH	Pharmaceuticals, antidepressants, personal care products	Lake	200 mg	Recoveries discussed in the paper in different seasons	[307]
**POCIS HLB** **PES membrane**	35 APIs and endocrine disruption	2014	Singapore	-	Pharmaceuticals	Tropical Lake flow ~3–5 cm/S	60 mg	Atorvastatin and norfluoxetine, from 52 to 196% (109 ± 32%), the 80–120% range for 26 of the compounds	[259]
**POCIS HLB** **PES membrane**	Pestisides, herbicides, fungicides and pharmaceuticals	2016	Canada	MeOH	Fungicides, herbicides, pharmaceuticals	Lake	200 mg	Atrazine was detected at all sites, and diuron, 2,4-D, and mecoprop were frequently detected. The fungicides carbendazim and thiophanate-methyl were detected at all sites, & a hydroxy-metabolite of the fungicide chlorothalonil was also widely detected	[257]
**POCIS HLB** **PES membrane**	25 pesticides	2018	Burkina Faso	MeOH, & MeOH/EtOAc(1:1, *v*/*v*), & EtOAc/Hx (1:4, *v*/*v*)	pesticides	Lake	200 mg	Atrazine, azadirachtin, carbofuran, chlorpyrifos, cypermethrin, dieldrin, imidacloprid, & profenofos exceeded 0.1 μg/L	[256]
**Oasis HLB** **PES membrane**	microcystin-LR (MC-LR)	2019	Canada	MeOH	Microcystin-LR	Lake	220 mg	The Rs 0.045 (±0.001) and 0.041 (±0.001) L per day for initial concentrations of 0.5 and 1.0 mg/L	[260]
**Oasis HLB** **PES membrane**	neonicotinoid insecticides NNIs, thiamethoxam, clothianidin and imidacloprid, Atrazine, 2,4-D, dicamba, carbendazim, thiophanate methyl and several azoles	2019	Canada	MeOH	Pesticides	Lake	200 mg	NNIs, thiamethoxam, clothianidin and imidacloprid 0.23 μg/L, Atrazine, 2,4-D, dicamba, carbendazim, thiophanate methyl and several azole-based fungicides were also widely detected	[255]
**Oasis HLB**	Pesticides	2019	Tunisia	ACN	Pesticides	Lagoon watershed		The results in different sites are reported in the paper	[308]

**Table 10:** Application of POCIS on drinking water.

POCIS Resins	Analyte	Year	Country	Elute	Application	Deployment Condition	Adsorbent Quantity	Analyte %Recoveries	Ref
**Oasis MAX, HRX, HLB, PES membrane**	Acidic herbicides	2012	France	MeOH, & MeOH/EtOAc 5:5 (*v*/*v*)	Pesticide	Drinking water	-	POCIS-MAX showed no influence of nitrates. MAX sorbent >82% recoveries	[265]
**Oasis HLB, PES membrane**	Eight alkylphenols, nine hormones, 11 pesticides, 27 pharmaceuticals and one UV filter	2013	France	MeOH, & MeOH: DCM 50:50	Alkylphenols, hormones, pesticides, pharmaceutical, UV filters	Tap water (using external thermostat tank)	200 mg	Details discussed in the paper	[266]
**POCIS HLB**	Carbamazepine, trimethoprim, sulfamethoxazole, ibuprofen, gemfibrozil, estrone and sucralose	2014	Canada	MeOH	Pharmaceuticals	Drinking water	220 mg	After 10 days: CBZ 894.7 ± 37.2 ng/L, Ibuprofen: 262.4 ± 80.1 ng/L, Gemfibrozil: 144.5 ± 31.3 ng/L, TPM: 8.7 ± 2.2 ng/L, Sucralose: 186.1± 15.0 ng/L	[309]
**POCIS HLB, PES membrane**	Atrazine-d5, caffeine-13C3, cotinine-d3, DIA-d5, fluoranthene-d10, lindane	2016	USA	Acetone, DCM	Pesticides, polycyclic, aromatic hydrocarbons, personal care products	Tap water	200 mg	4.51 ± 0.34 g/g (atrazine-d5), 4.62 ± 0.30 g/g (caffeine-13C3), 4.01 ± 0.08 g/g (cotinine-d3), 3.87 ± 0.24 g/g (DIA-d5), 4.42 ± 0.16 g/g (fluoranthene-d10), and 4.65 ± 0.14 g/g (lindane).	[264]
**POCIS HLB, Additionally, DOWEX, PES membrane**	34 pesticide, personal care products and hydrocarbons	2016	USA	Acetone, & DCM,	Pesticides, polycyclic aromatic hydrocarbons, personal care products	Tap water	200 mg	Recoveries average: Dowex Optipore L-493: 90% (range: 66–127%), HLB: 91% (range: 66–135%), and Osorb media: 96% (range:63–127%)	[263]
**POCIS HLB, PES membrane**	73 compounds	2016	Norway	MeOH	Pharmaceuticals, endocrine disrupting substances, pesticides, herbicides, drugs of abuse	Drinking water	200 mg	Results for prediction model discussed	[262]
**POCIS HLB, PES membrane**	Diclofenac (DIC), ketoprofen (KET), mefenamic acid (MEF), naproxen (NAP), ibuprofen (IBU), ketoprofen-d3 (KET-d3), perfluorooctanoic acid (PFOA), perfluorooctanesulfonate (PFOS) and Caffeine (CAF)	2018	Italy	Acetone	Pharmaceuticals, perfluorinated compounds, caffeine	Drinking water in vitro	200 mg	Caffeine: 0.07–0.93 ng/L, perfluorinated compounds: 2.93–13.42 ng/L	[267]
**POCIS HLB, PES membrane**	Imidacloprid, clothianidin, thiamethoxam, acetamiprid, thiacloprid, a hydroxy metabolite	2018	Canada	MeOH: Acetone 60:40 *v*/*v*	Pesticides	Drinking water	220 mg	Clothianidin 300 µg/L imidaclopid 500 µg/L thiamethoxam 5 µg/L	[261]
**Oasis HLB**	Microcystins risk assessment	2019	Czech Republic	-	Microcystins	Drinking water reservoir Depth: 13, 28, 46 m, flow velocities ranging between 0.01 and 0.15 m/s	90 mg	20–200 pg/L after 14-d deployment and 1–12 ng/L	[268]
**POCIS Oasis WAX, PES membrane**	26 per- and polyfluoroalkyl substances (PFASs)	2019	Sweden	MeOH	Per, polyfluoroalkyl substances (PFASs)	Drinking water in treatment plant	200 mg	64–89%	[310]

### 4.4. Application of POCIS, In Vitro Laboratory Studies

The efficiency of POCIS has been assessed in vitro under laboratory simulated conditions for the evaluation of a range of pharmaceuticals, pesticides and beta-blockers using different amount of sorbent (Table 11). Oasis HLB (200 mg) of sorbent was applied to moderate polarity pesticides and their metabolites such as propiconazole (log K_ow_ = 3.72) and tebuconazole (log K_ow_ = 3.7) and showed a low efficiency of POCIS in adsorbing these compounds, which were initially at low concentrations in the aquatic environment of study [311]. Additionally, ten pharmaceutical compounds ranging between log K_OW_ 0.16 to 4.51 (0.16_log K_OW__4.51) under laboratory conditions were studied to evaluate the effect of flow velocity on the accuracy of passive sampling [312]. Using the POCIS with PRC (Performance Reference Compounds) approach and o-DGT, these absorption systems were applied under different water velocities (2 < V < 18 cm/s), to evaluate the sampling of atenolol, carbamazepine, diclofenac, fluoxetine, ketoprofen, metoprolol, paroxetine, propranolol, sulfamethoxazole, and trimethoprim. Both samplers were able to limit the flow effects ensuring the accuracy of POCIS performance (within 20% uncertainty). In addition, even though o-DGT was more efficient in quiescent (dormant) situations it has less sensitivity compared to POCIS, at adsorbing these analytes [312].

In the in vitro laboratory scale studies, unlike the other applications for POCIS, the most dominant studies have reported higher amounts of HLB sorbent efficiency (>200 mg). Ibrahim et al. have used 220 mg of Oasis HLB resin for a range of 17 polar pesticides (1.15 ≤ log K_ow_ ≤ 3.71) during a 15-day study where the sampling rate ranged between 67.9 to 279 mL/day increased with increasing hydrophobicity of the pesticides [313]. In addition, Fauvelle et al., presented a study on the comparison of the capacity of different amounts of Oasis HLB (200 and 600 mg) in a pharma-POCIS device on the uptake of acidic (2,4-dichlorophenoxyacetic acid, (ESA), acetochlor oxanilic acid, bentazon, dicamba, mesotrione, and metsulfuron) and polar herbicides such as (atrazine, diuron, and desisopropylatrazine). According to these studies 200 mg Oasis HLB is not efficient in absorbing compounds with high acidity and polarity because of their fast thermodynamic equilibrium with the HLB sorbent. Separately, 600 mg of sorbent showed a sampling rate two times higher compared with 200 mg over 35 days [239].

The effects of water temperature on the accumulation of 48 pesticides, insecticides and fungicides on POCIS using 220 mg of Oasis HLB sealed between hydrophilic microporous polyethersulfone (PES) membranes for 28 days was investigated [314]. The results demonstrated that increasing the water temperature led to an increasing sampling rate, reported at 18, 24, and 30 °C ranged from 0.00676 to 0.262, 0.00603 to 0.312, and 0.00426 to 0.603, respectively [314]. Togola et al. evaluated the application of POCIS for pharmaceutical monitoring under environmental conditions such as salinity, temperature, and pollutants. Applying three conditions for Rs (0 PSU/21 °C, 35 PSU/21 °C and 0 PSU/15 °C), showed average recoveries for caffeine of 1622 ng/L, amitriptyline 355 ng/L, doxepin 253 ng/L, imipramine 377 ng/L, carbamazepine 226 ng/L, diazepam 435 ng/L, nordiazepam 629 ng/L and ibuprofen 1128 ng/L. This study showed the applicability of POCIS in the detection of trace concentration of compounds below the detection limit of discrete (non-continuous) sampling approaches [315]. Five different types of POCIS-SR, POCIS-A, POCIS-B, chemcatcher PRS, chemcatcher C18 were tested for the determination of 124 different pesticides in water under simulated laboratory ambient (20 °C) temperature and 10 cm/s turbulence conditions. POCIS-SR showed a better capability in the up-take of hydrophobic compounds (log K_ow_ > 5.3) whereas the other devices (POCIS-A, POCIS-B, chemcatcher PRS, chemcatcher C18) showed better results for hydrophilic compounds (log K_ow_ < 0.7) [316].

**Table 11:** Application of POCIS, in vitro laboratory scale.

POCIS Resins	Analyte	Year	Country		Elute	Application		Deployment Condition	Adsorbent Quantity	Analyte %Recoveries	Ref
**Oasis HLB, PES membrane**	Caffein, Amitripthiline, Doxepine, Imipramine, Carbamazapine, Diazepam, Nordizepam, Ibuprofen, Gemfibrozile, Naproxine, Diclofenac, Ketoprofen	2007	France		EtOAc/Ace 50/50 *v*/*v*	Pharmaceuticals		Laboratory simulation	200 mg	Average recoveries: Caf: 1622 ng/L, Ami: 355 ng/L, Dox: 253 ng/L, Imi: 377 ng/L, Cbz: 226 ng/L, Dzp: 435 ng/L, Ndzp: 629 ng/L, Ibu: 1128 ng/L, Gem: 1744 ng/L, Nap: 673 ng/L, Diclo: 606 ng/L, Keto: 388 ng/L	[315]
**Oasis HLB, PES membrane**	Range of substances in different pH reported	2011	Canada		MeOH	Pharmaceuticals, personal care products, disrupting substances		Laboratory scale River and Tap water (water chamber in lab)	200 mg	Recoveries in different pH are reported	[174]
**Oasis HLB, PES membrane**	Atrazine, simazine, desethylatrazine (DEA), desisopropylatrazine (DIA), desethylterbuthylazine (DET), terbuthylatrazine, diuron, isoproturon, chlortoluron, linuron, propyzamide, alachlor, metolachlor, acetochlor, metalaxyl, penconazole, and azoxystrobine	2013	France		ACN	pesticide		Laboratory calibration experiment	230 mg	Sampling rate: 67.9–279 mg/L	[313]
**Pharma-POCIS Oasis HLB PES membrane**	Polar pesticides and metabolites	2013	France		ACN	pesticide		Laboratory in situ sampling	200 mg	169 to 479 mL/g day	[311]
**POCIS HLB, Nylon membrane**	A wide range of pharmaceuticals and pesticides	2014	France		MeOH, DCM/MeOH (50:50; *v*/*v*), and DCM	Pesticides, pharmaceuticals		Laboratory water samples	200 mg	Results discussed in the paper	[317]
**Strata XAW, PES membrane**	Perfluorohexanoate (PFHxA), perfluoroheptanoate (PFHpA), perfluorooctanoate (PFOA), perfluorononanoate (PFNA), perfluorodecanoate (PFDA), perfluoroundecanoate (PFUnDA)	2014	Australia		0.1% (*v*/*v*) ammonia MeOH, & MeOH	Perfluorinated chemicals (PFCs)		Laboratory water sample	600 mg	PFPeA 0.078 ± 0.02 L/d, PFHxA 0.118 ± 0.01 L/d, PFNA 0.165 ± 0.004 L/d, PFHxS 0.182 ± 0.01 L/d	[214]
**POCIS HLB, PES membrane**	2,4-dichlorophenoxyacetic acid (2,4-D), acetochlor ethanesulfonic acid (ESA), acetochlor oxanilic acid, bentazon, dicamba, esotrione, and metsulfuron, atrazine, diuron, esisopropylatrazine herbicides	2014	France		MeOH & MeOH/EtOAc 50: 50 (*v*/*v*)	Herbicides		Laboratory water samples	200, 600 mg	Increasing sorbent to 600 mg resulted in sampling rates (Rss) twice as high as those observed with 200 mg	[239]
**POCIS (5 different types) SR, POCIS-A, POCIS-B, Chemcatcher RPS, Chemcatcher C18**	124 pesticides	2015	Sweden		MeOH, & DCM/MeOH (8/2, *v*/*v*)	Pesticides		Laboratory condition	220 mg	Results for different POCIS devices discussed	[316]
**Pharma-POCIS HLB** **PES membrane**	20 pesticides, insecticides, herbicides	2016	Japan		EtOH	Pesticides		Laboratory pesticide sample water	220 mg	Sampling rate increased at 18 °C from 0.00676 to 0.262, 24 °C 0.00603 to 0.312, 30 °C 0.00426 to 0.603.	[314]
**Carbon nanotubes, PES membrane**	Carbamazepine, diclofenac, β-estradiol, p-nitrophenol, 3,5-dichlorphenol, sulfapyridine, sulfamethoxazole	2017	Poland		ACN/MeOH/DCM, (40:40:20; *v*/*v*),	Pharmaceuticals, phenols		Laboratory water sample	100 mg	Sulfapyridine: 79.8 ± 0.2%, Sulfamethoxazole: 41.5 ± 0.1% Carbamazepine: 96.6 ± 1.5% p-nitrophenol: 70.5 ± 0.1% 17-β-estradiol: 77.1 ± 0.5% 3,5-dichlorophenol: 103.1 ± 1.8% diclofenac: 76.3 ± 1.4%	[318]
**Oasis HLB** **PES membrane**	Atenolol, cabamazapine, Diclofenac, Fluoxetine, Ketoprofen, Metoprolol, Paroxetine, Propaonalol, Sulfamethaxazole, Trimethoprime	2019	France		MeOH, & MeOH/ EtOAc, 75:25 *v*/*v*	Pharmaceuticals		Ultrapure water	200 mg	Effect of flow velocities is assessed (2 < V < 18 cm/s)	[312]
**POCIS Oasis HLB**	44 pharmaceuticals	2020	France		MeOH & 75:25 (*v*/*v*) MeOH: EtOAc	Pharmaceuticals pump (flow rate = 13 m^3^/h)		laboratory-scale artificial river	200 mg	Econazole, fenbendazole, fenofibrate, metformin, thioridazine, and triclabendazole) were not sampled by POCIS and 12 compounds are not available	[319]
**POCIS HLB** **PES membrane**	neonicotinoid pesticides	2020	Japan		MeOH: ACE 2:1	neonicotinoid pesticides		Laboratory scale	20 mg	Suitable for neonicotinoid detection in lower concentration	[320]

Abbreviations: EtOAc (Ethyl acetate), Ace (Acetone), MeOH (Methanol), CAN (Acetonitrile), DCM (Dichloromethane), Ammonia MeOH (Methanolic ammonia), EtOH (Ethanol).

## 5. Conclusions

In this review, scientific literature associated with two in situ methods, Solid Phase Adsorption Toxin Tracking (SPATT) and Polar Organic Chemical Integrative Sampler (POCIS), for the collection and concentration of biotoxins and pharmaceuticals in environmental waters, has been investigated. The application of Solid Phase Adsorption Toxin Tracking (SPATT) and Polar Organic Chemical Integrative Sampler (POCIS) to pre-concentrate a range of marine toxins, pesticides and pharmaceutical compounds that occur at low levels in marine and environmental waters has been critically discussed and summarised in tabular format

A variety of adsorption substrates in SPATT and different sorbents in POCIS were reviewed. Laboratory and field studies demonstrated the efficacy and ability of SPATT technology as reliable in situ methods to absorb a range of lipophilic and hydrophilic marine biotoxins, pharmaceuticals, pesticides, antibiotics and microcystins in marine water, freshwater and wastewater ecosystems.

Furthermore, analytical methods such as TLC, LC-MS and LC-MS/MS that had been used to detect the biotoxins of different toxin classes OA/DTXs, PTXs, YTXs, AZAs DA, have been addressed.

The review shows that previous studies primarily focused on adsorption and desorption efficiencies; this leaves gaps in the knowledge regarding quantitative sampling and isotherm characterization in relation to specific biotoxins. Studies showed that although HP20 is successful in the adsorption of a range of lipophilic and hydrophilic toxins, it has relatively slow uptake, however the efficiency of HP20 in adsorption of toxins is highest compared to other aromatic sorbents due to its large pore size. These studies emphasise the importance of knowledge gathering and experimentation to determine the duration that HP20 remains in the integrative phase.

The maximal capacity of HP20 is relatively higher than SP700 that means pore size plays an important rule is adsorption efficiency along with the polarity of toxins. The average recoveries of lipophilic and some hydrophilic toxins with HP20 is around 90% and SP700 shows 69–72%. Average recoveries of lipophilic toxins for HP20, Strata-X and Oasis HLB after 24 h shows 70, 50 and 40%. POCIS using oasis HLB shows an average recovery between 80–120% recoveries for APIs and endocrine disruption. Finally, this review examines the marine toxin area categorising the main toxin groups, according to toxicity and aetiology, and historically reviews the application of SPATT as an early warming strategy for marine toxin surveillance.

## Figures and Tables

**Table 3:** Extraction steps for most common resins.

SPATT Resin	Toxin Groups	Elute	References (Refs.)
**DIAION HP20, SEPABEADS SP825L, SP850 and SEPABEADS SP700**	Microcystins	50% MeOH	[6,165]
**DIAION HP20, SP 207, HP2MG**	PTX, PTX 2 SA, PTX 11, PT11 SA, OA, OA-ester, YTX	MeOH	[5,156]
**DIAION HP20**	DA, CTXs	MeOH	[5,171,177]
		Ammonium acetate in 50% MeOH	
		Ammonium acetate in 50% MeOH	
**DIAION HP20**	Cyanotoxins, Okadaic acid, Saxitoxin and related PSTs	MeOH	[156]

**Table 4:** Extraction steps for modified resins [2,156].

SPATT Phases	Elute 1	Elute 2
Strata-X (200 mg)	100% MeOH	-
PAC G-60 (200 mg)	5% Formic acid in 70% MeOH	5% conc. HCL in MeOH (6 mL)
AG 50W-X4 (200 mg)	5% NH_3_ in MeOH (5 mL)	-
Amberlite IRP-64 (200 mg)	5% Formic acid in 70% MeOH	-

## Supplementary Materials

The following supporting information can be downloaded at: https://www.mdpi.com/article/10.3390/molecules27227898/s1: Marin Biotoxins Figure S1: Marine biotoxin structures. (a) okadaic acid, (b) yessotoxin, (c) azaspiracid, (d) saxitoxin, (e) domoic acid, (f) Brevetoxin (PbTX-1) Type-A, (g) PbTX-2 (Type-B), (h) PbTX-3(Type-B), (i) Ciguatoxin-1 (CTX), (j) Ciguatoxin-2, (k) Ciguatoxin-3, (l) Ciguatoxin-4A, (m) Ciguatoxin-4B, (n) Pectenotoxin-1 (PTX-1), (o) Pectenotoxin-2 (PTX-2), (p) Dinophysistoxin 1 (DTX-1), (q) Dinophysistoxin 2 (DTX-2), (r) Dinophysistoxin 3 (DTX-3). Figure S2: (a) SPATT bags and discs with various resins contained within 80 mm polyester mesh; (b) SPATT bags being deployed. However, after this initial design other studies applied the same bags in a different manner to support the SPATT bags in the water flow. Diagram [160]; (c) showing SPATT bags among holding tubes. (A) 100 mm nylon mesh, (B) resin, (C) inner holding ring, (D) outer holding ring, (F) 75 mm diameter embroidery ring and (E) final assembled sampling disk [181]. SPATT bags attached to aluminum alloy [162]. Figure S3: (a) Chemical Structure DIAION HP-20 (Aromatic synthetic adsorbent ion-exchange resin) Styrene-divinylbenzene [164]; (b) SEPABEADS SP700 (Aromatic synthetic adsorbent ion-exchange resin) [167] (c) SEPABEAD SP207 (Modified Aromatic synthetic adsorbent ion-exchange resin) Brominated styrene—divinylbenzene [166] (d) DIAION HP2MG (Methacrylic synthetic adsorbent ion-exchange resin) Polymethacrylate [166]. Figure S4: Schematic diagram of pumping system [161]. Figure S5: Organic Chemical Integrative Sampler (POCIS) device [321]. (a) POCIS or Aquasense-P disk; (b) Polar Organic Chemical Integrative Sampler (POCIS) carrier; (c) carrier on which one to three POCIS can be mounted. (d) schematic diagram of extraction of analyte from POCIS device.
