# Supplementary material for: A Review of In Situ Methods—Solid Phase Adsorption Toxin Tracking (SPATT) and Polar Organic Chemical Integrative Sampler (POCIS) for the Collection and Concentration of Marine Biotoxins and Pharmaceuticals in Environmental Waters
Source: Molecules. 2022 Nov 15;27(22):7898. doi: 10.3390/molecules27227898 (PMC9698218; doi:10.3390/molecules27227898)
Supplement: Supplementary file 1 [file molecules-27-07898-s001.zip › molecules-1815524-supplementary.pdf]

Supplementary Materials

Contents

Figure S1: Marine biotoxin structures: (**a**) Okadaic acid (DSP); (**b**) yessotoxin (DSP); (**c**) Azaspiracid (AZP); (**d**) Saxitoxin (PSP); (**e**) Domoic acid (NSP); (**f**) 7 Brevetoxin (PbTX-1) Type-A (NSP); (**g**) PbTX-2 (Type-B); (**h**) PbTX-3(Type-B); (**i**) Ciguatoxin-1 (CTX); (**j**) Ciguatoxin-2; (**k**) 8 Ciguatoxin-3; (**l**) Ciguatoxin-4A; (**m**) Ciguatoxin-4B; (**n**) Pectenotoxin-1 (PTX-1: DSP), (**o**) Pectenotoxin-2 (PTX-2: DSP), (**p**) Dinophysistoxin-1 (DTX-1: DSP), (**q**) Dinophysistoxin-2 (DTX-2: DSP) and (**r**) Dinophysistoxin-3 (DTX-3: DSP).

Figure S2: (**a**) SPATT bags and discs with various resins contained within 80 mm polyester mesh; (**b**) SPATT bags being 12 deployed. However, after this initial design other studies applied the same bags in a different manner to support the 13 SPATT bags in the water flow. Diagram [158]; (**c**) SPATT bags among holding tubes. (A) 100 mm nylon mesh, 14 (B) resin, (C) inner holding ring; (D) outer holding ring, (F) 75 mm diameter embroidery ring and (E) final assembled 15 sampling disk [242]. (**d**) SPATT bags attached to aluminum alloy [160].

Figure S3: (**a**) Chemical Structure DIAION HP-20 (Aromatic synthetic adsorbent ion-exchange resin) Styrene-18 divinylbenzene [162]; (**b**) SEPABEADS SP700 (Aromatic synthetic adsorbent ion-exchange resin) [165] (**c**) SEPABEAD 19 SP207 (Modified Aromatic synthetic adsorbent ion-exchange resin) Brominated styrene divinylbenzene [164]; (**d**) 20 DIAION HP2MG (Methacrylic synthetic adsorbent ion-exchange resin) Polymethacrylate [164].

Figure S4: Schematic diagram of pumping system [159].

Figure S5: Organic Chemical Integrative Sampler (POCIS) device [316]: (**a**) POCIS or Aquasense-P disk; (**b**) Polar 25 Organic Chemical Integrative Sampler (POCIS) carrier; (**c**) carrier on which one to three POCIS can be mounted; (**d**) 26 schematic diagram of extraction of analyte from POCIS device.

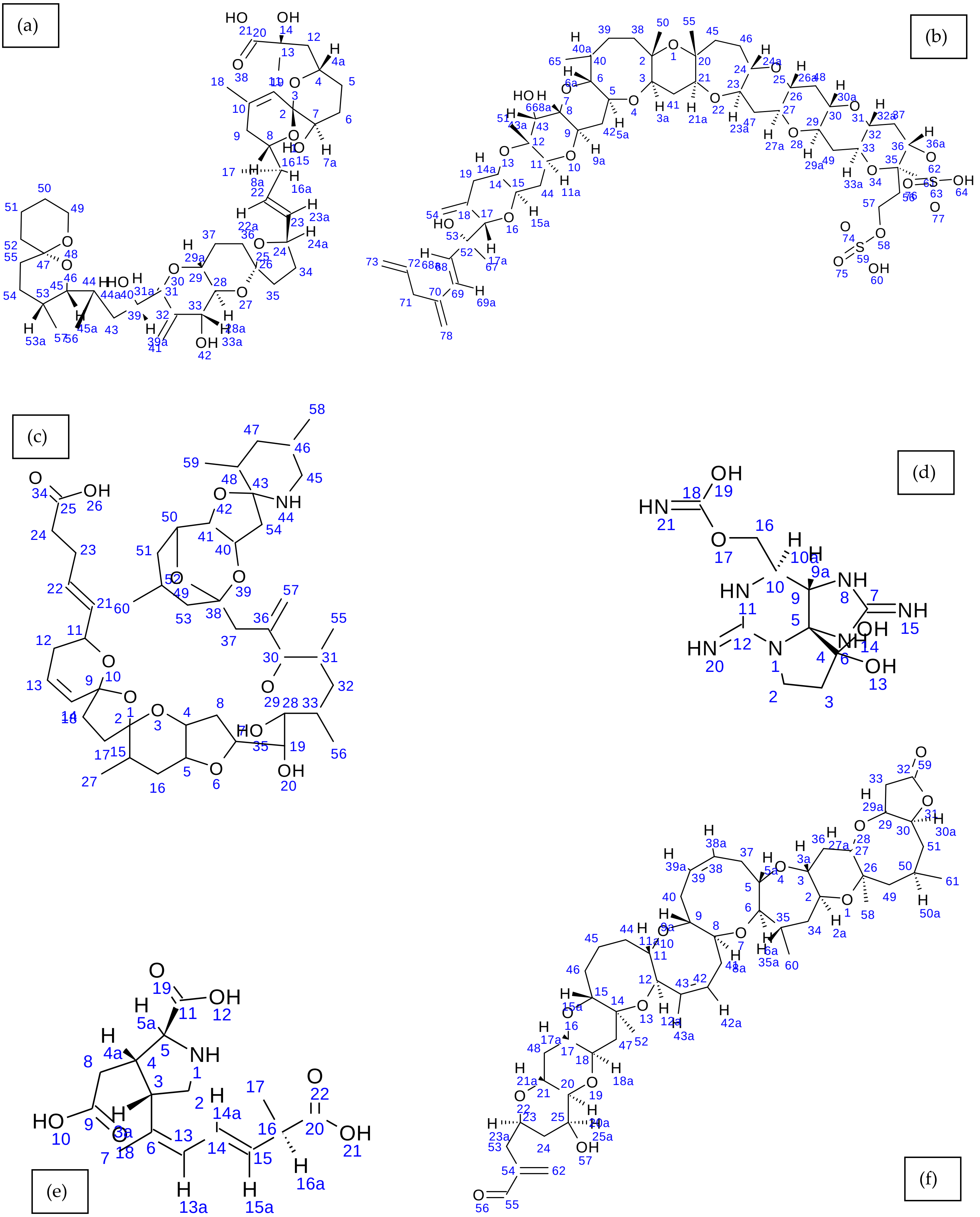
(a)
(b)
(c)
(d)
(e)
(f)

(g)

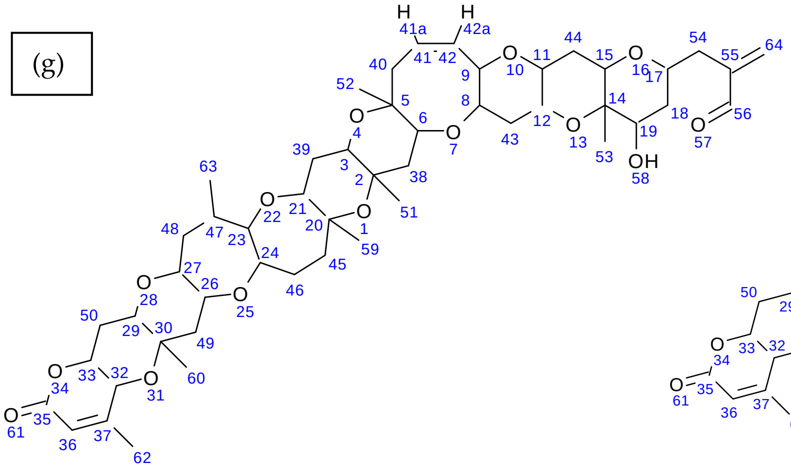

(h)

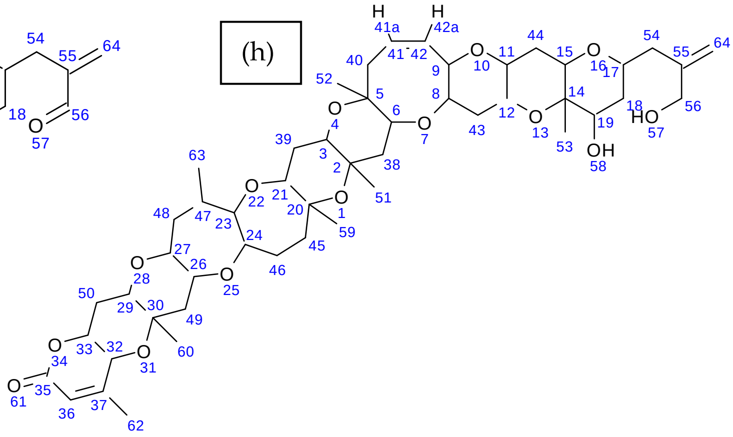

(i)

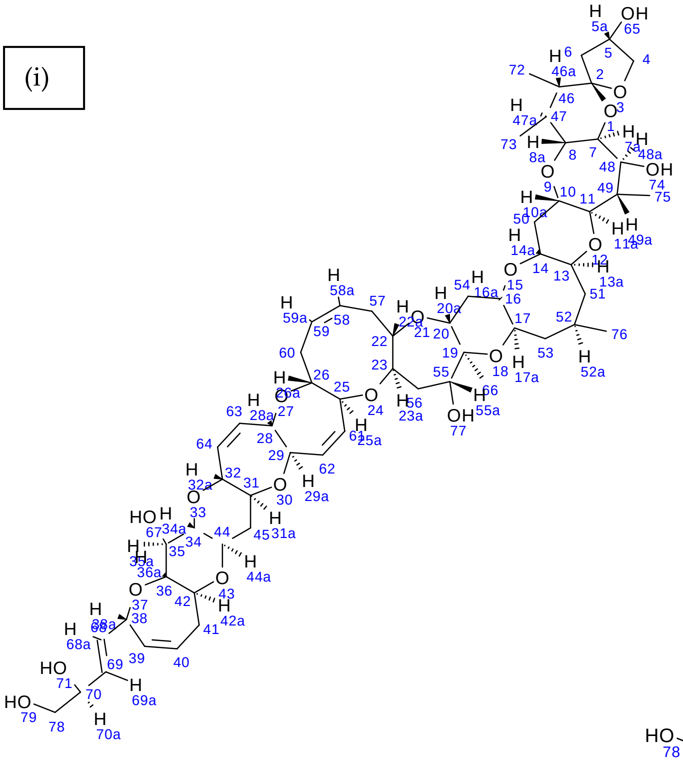

(j)

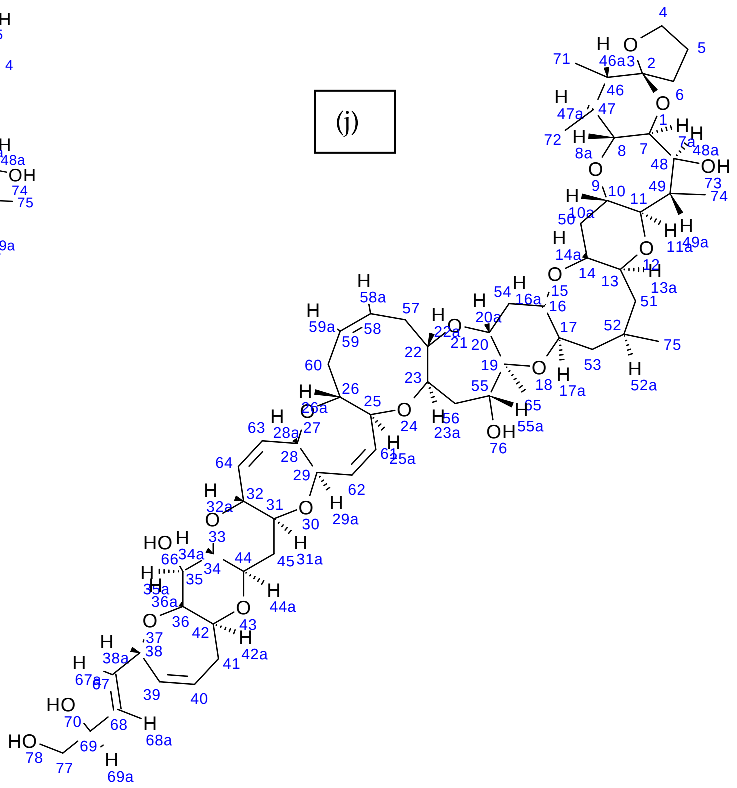

(k)

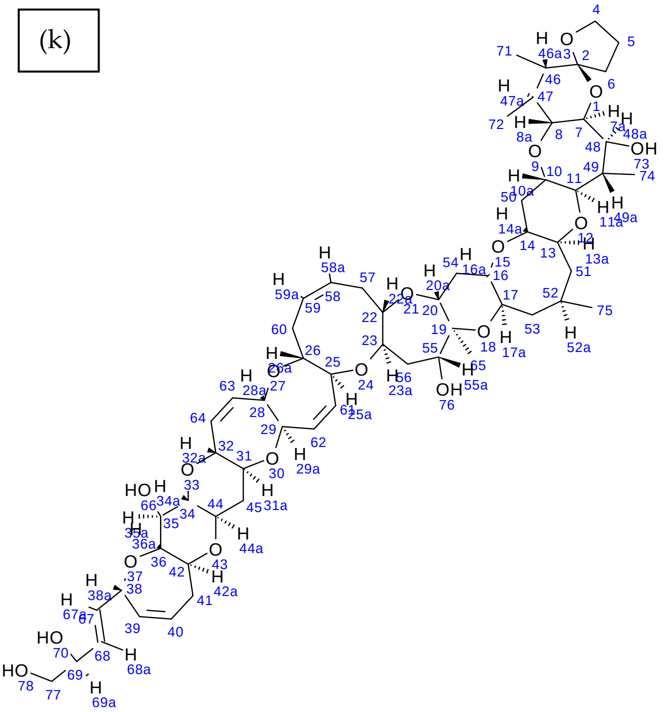

(l)

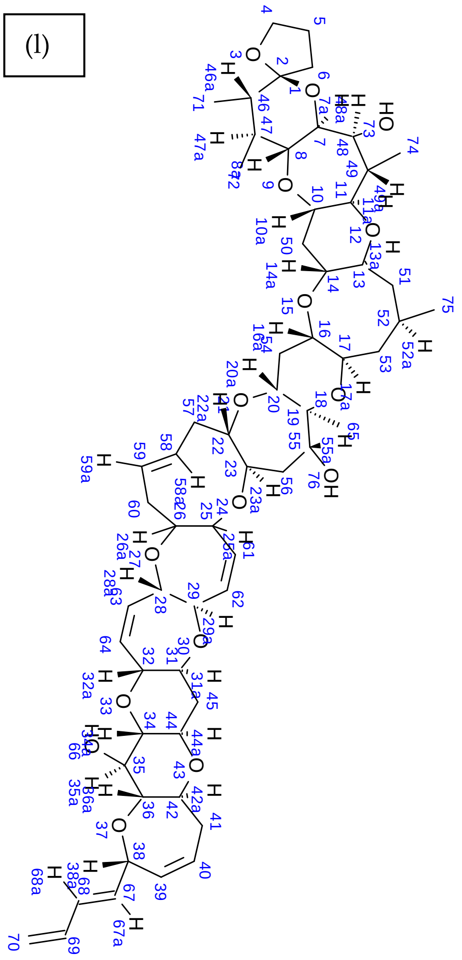

(m)

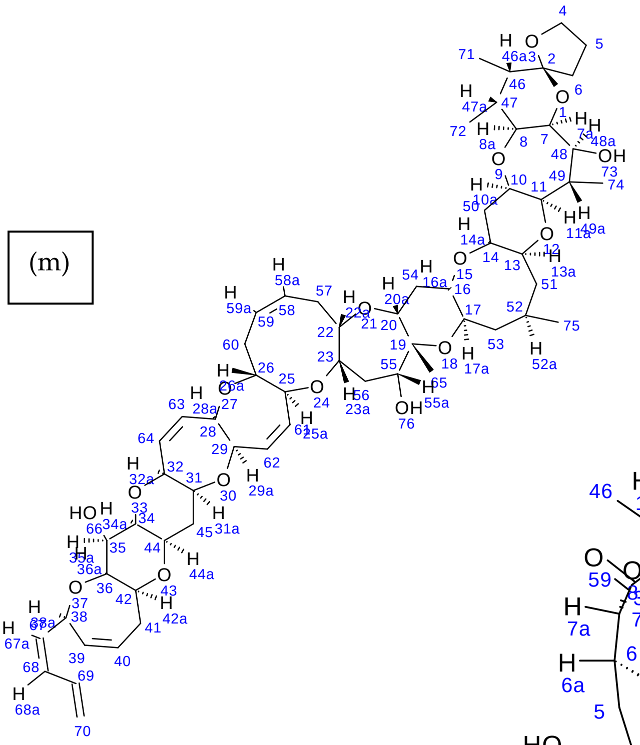

(n)

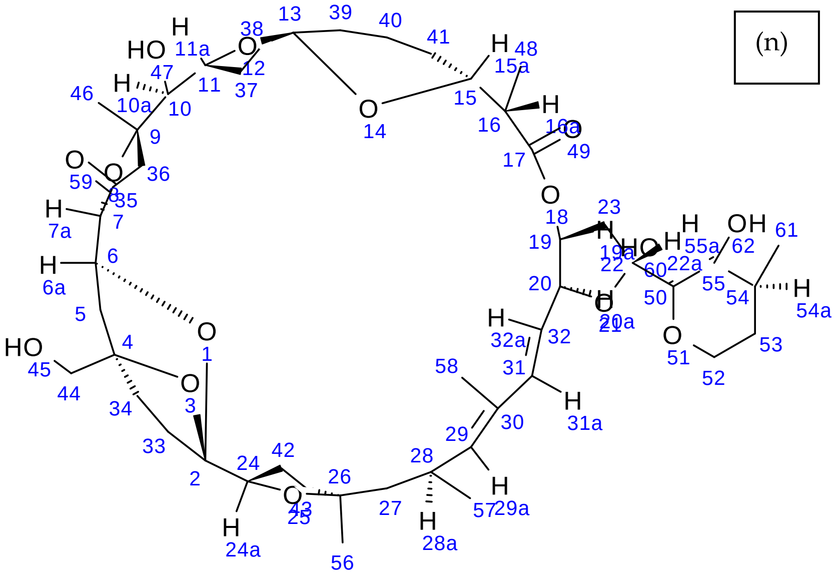

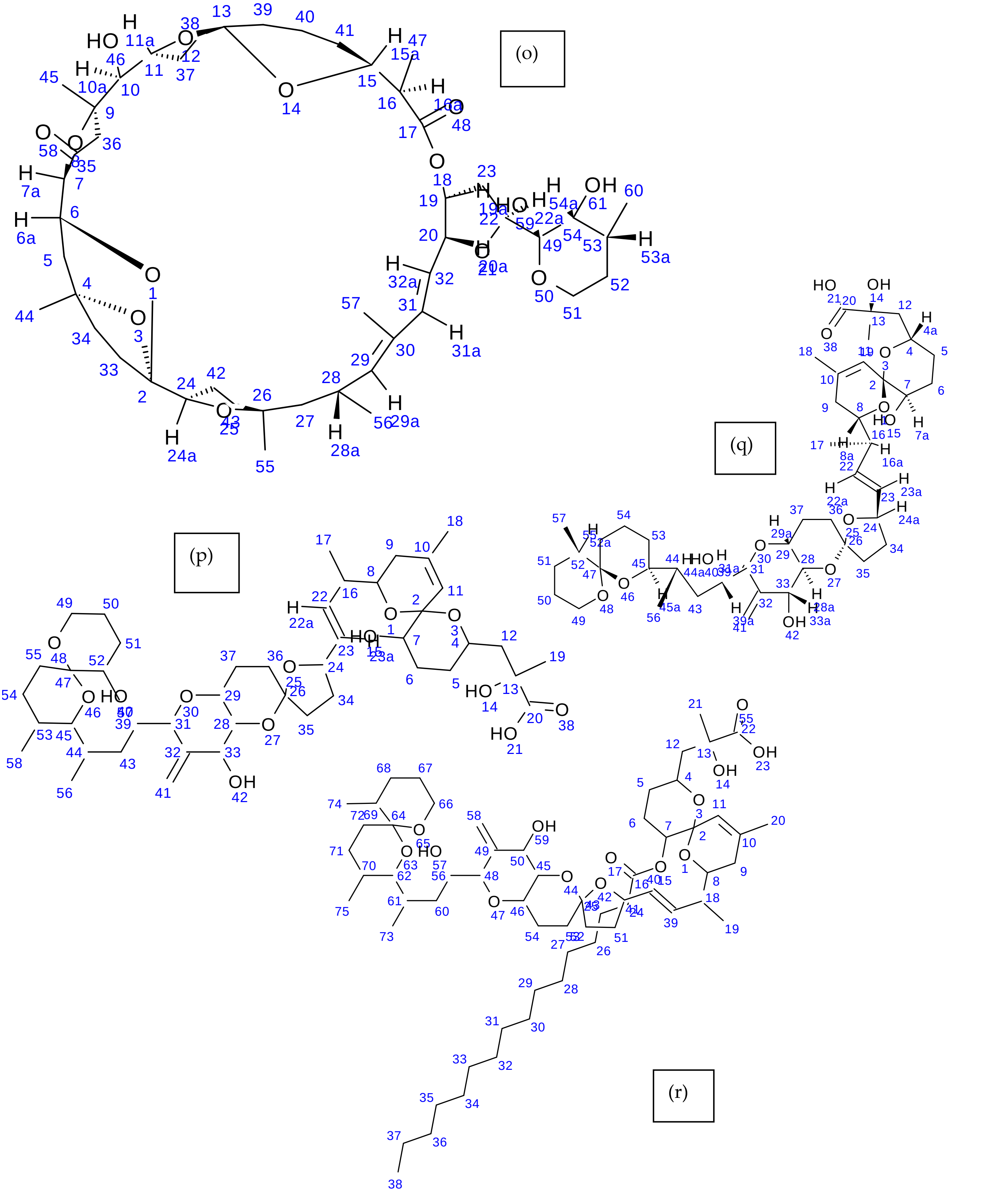

Figure S1. Marine biotoxin structures: (**a**) Okadaic acid (DSP); (**b**) yessotoxin (DSP); (**c**) Azaspiracid (AZP); (**d**) Saxitoxin (PSP); (**e**) Domoic acid (NSP); (**f**) 7 Brevetoxin (PbTX-1) Type-A (NSP); (**g**) PbTX-2 (Type-B); (**h**) PbTX-3(Type-B); (**i**) Ciguatoxin-1 (CTX); (**j**) Ciguatoxin-2; (**k**) 8 Ciguatoxin-3; (**l**) Ciguatoxin-4A; (**m**) Ciguatoxin-4B; (**n**) Pectenotoxin-1 (PTX-1: DSP), (**o**) Pectenotoxin-2 (PTX-2: DSP), (**p**) Dinophysistoxin-1 (DTX-1: DSP), (**q**) Dinophysistoxin-2 (DTX-2: DSP) and (**r**) Dinophysistoxin-3 (DTX-3: DSP).

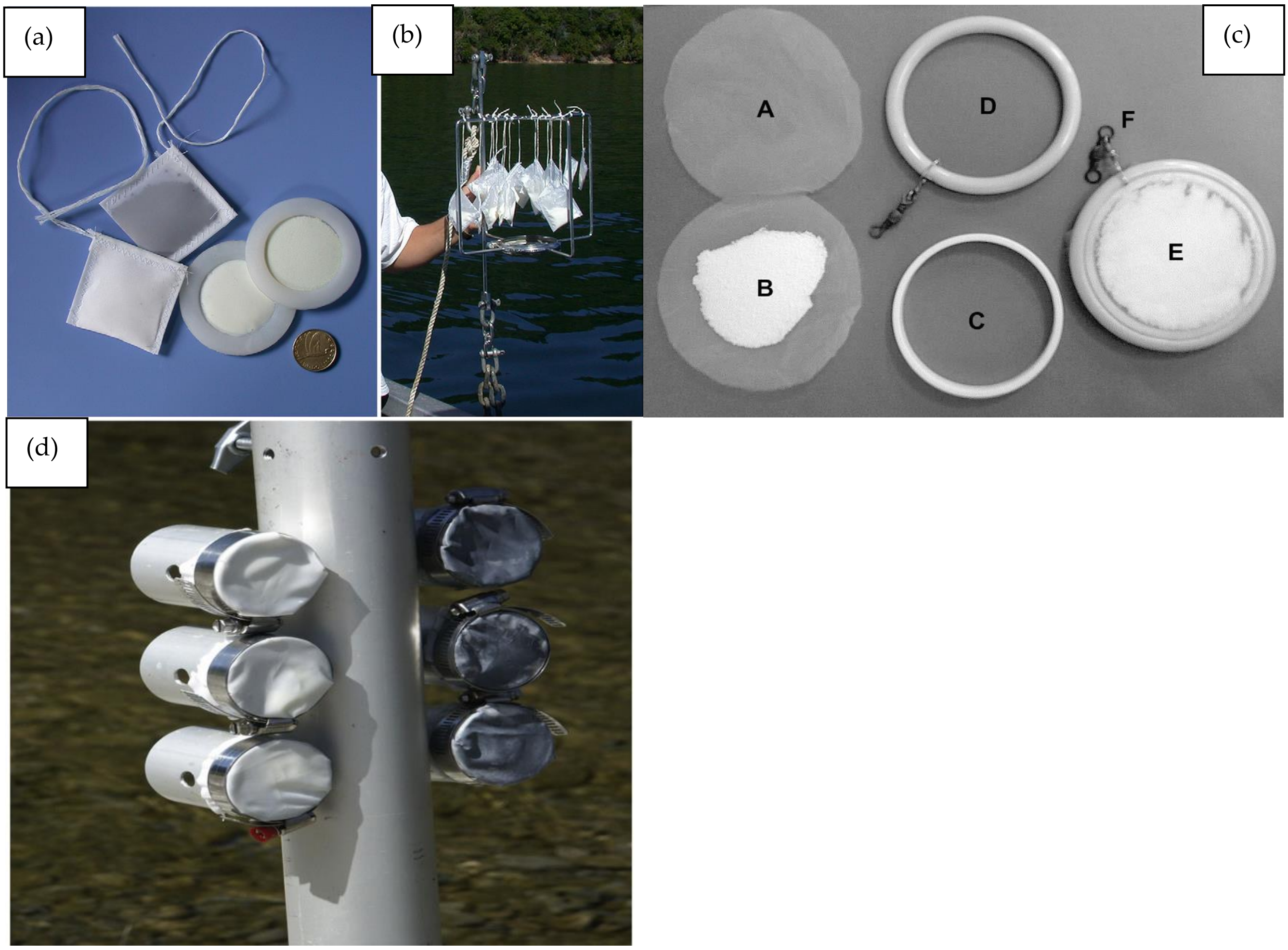

Figure S2. (**a**) SPATT bags and discs with various resins contained within 80 mm polyester mesh; (**b**) SPATT bags being deployed. However, after this initial design other studies applied the same bags in a different manner to support the SPATT bags in the water flow. Diagram [158]; (**c**) SPATT bags among holding tubes. (A) 100 mm nylon mesh, (B) resin, (C) inner holding ring; (D) outer holding ring, (F) 75 mm diameter embroidery ring and (E) final assembled sampling disk [242]. (**d**) SPATT bags attached to aluminum alloy [160].

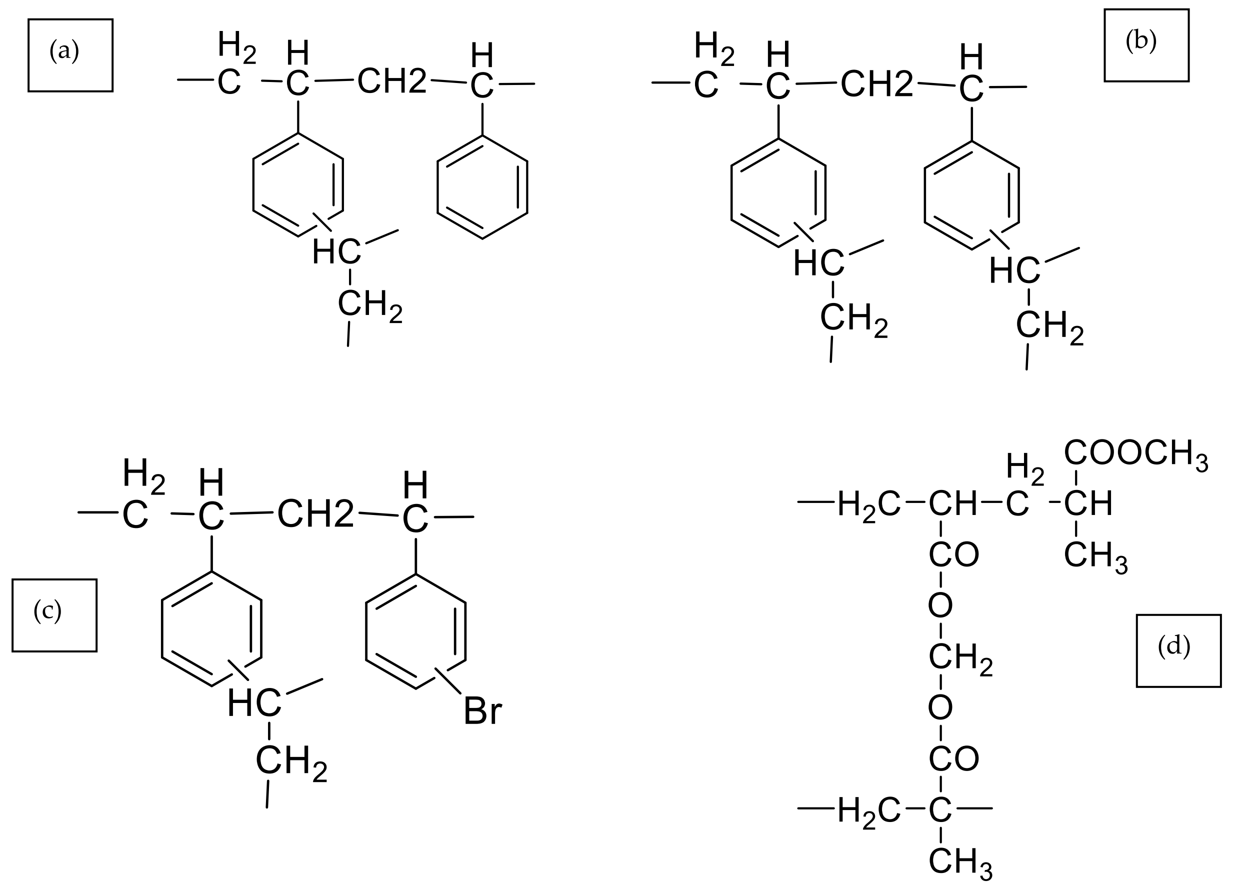

Figure S3. (**a**) Chemical Structure DIAION HP-20 (Aromatic synthetic adsorbent ion-exchange resin) Styrene-divinylbenzene [162]; (**b**) SEPABEADS SP700 (Aromatic synthetic adsorbent ion-exchange resin) [165] (**c**) SEPABEAD SP207 (Modified Aromatic synthetic adsorbent ion-exchange resin) Brominated styrene divinylbenzene [164]; (**d**) DIAION HP2MG (Methacrylic synthetic adsorbent ion-exchange resin) Polymethacrylate [164].

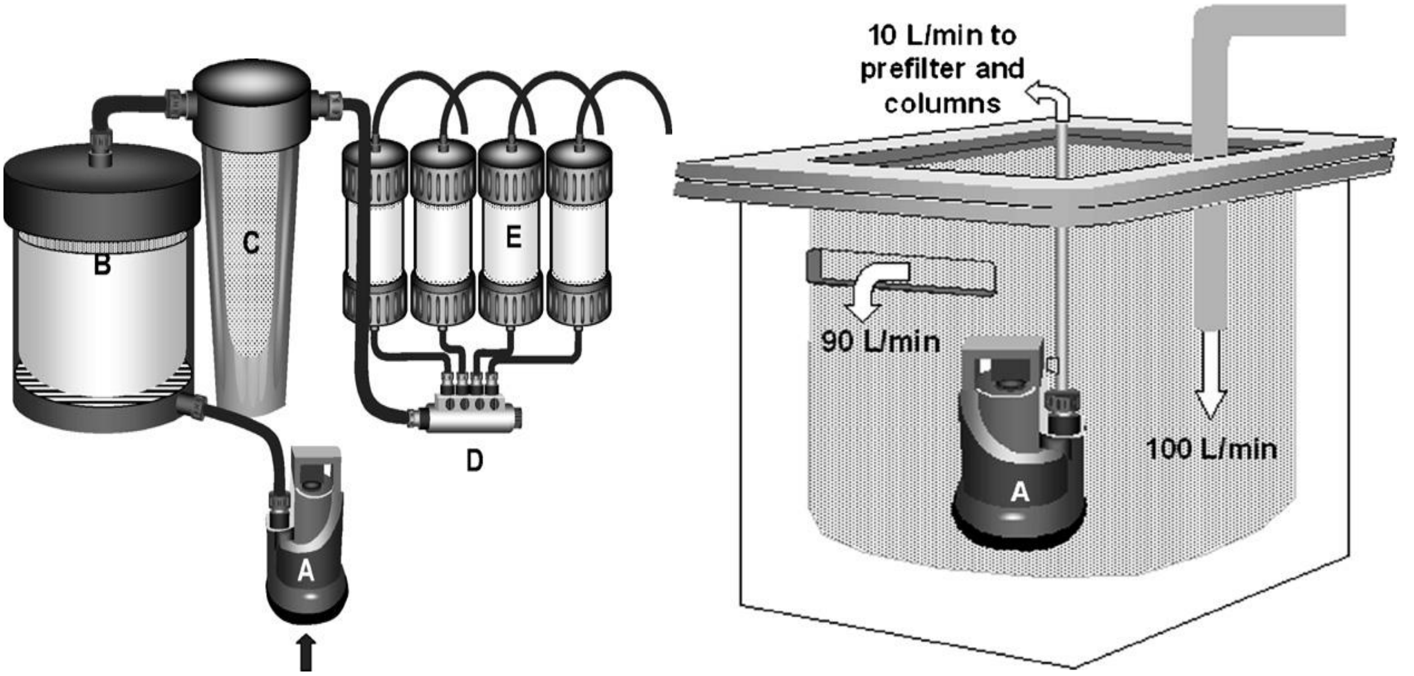

Figure S4. Schematic diagram of pumping system [159].

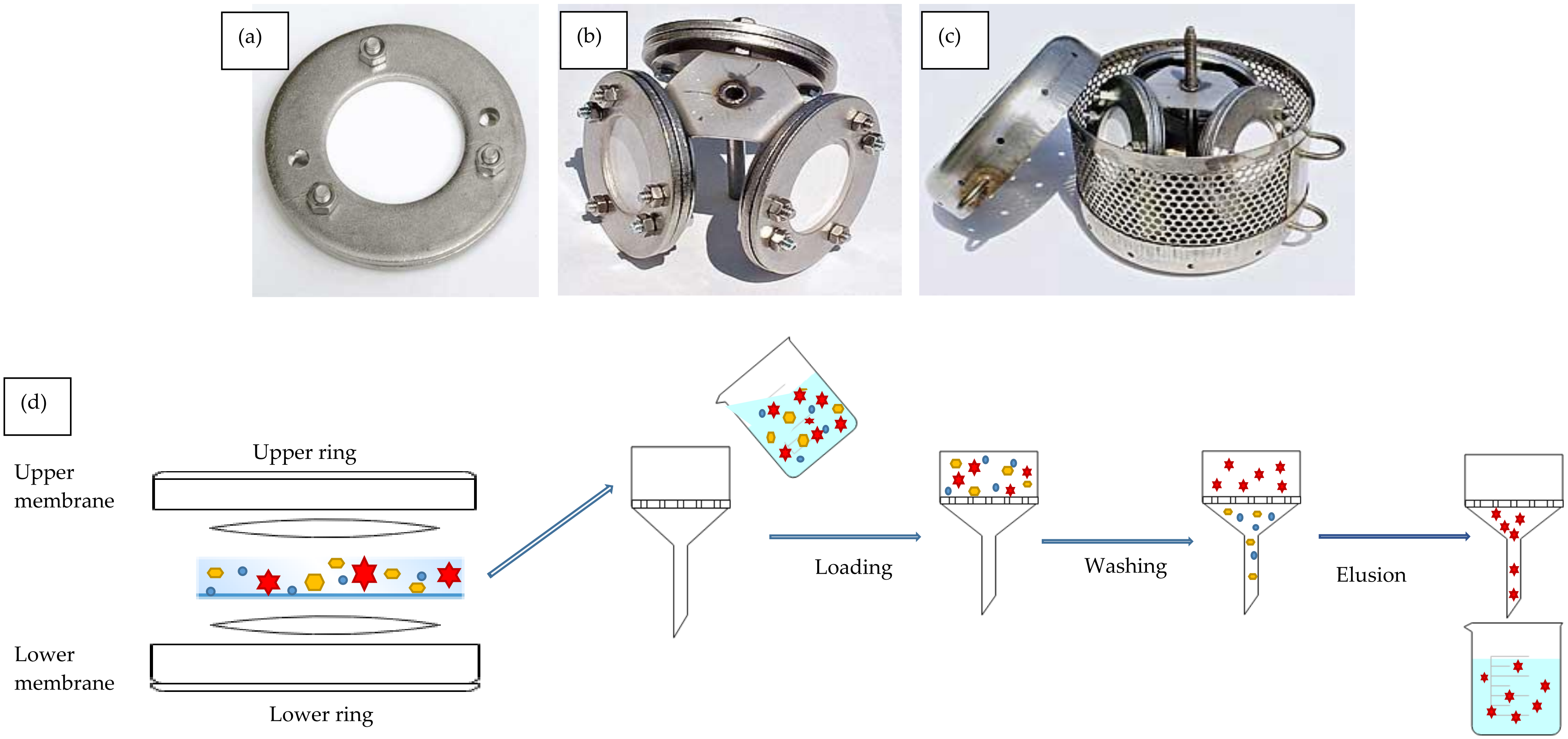

Figure S5. Organic Chemical Integrative Sampler (POCIS) device [316]: (**a**) POCIS or Aquasense-P disk; (**b**) Polar 25 Organic Chemical Integrative Sampler (POCIS) carrier; (**c**) carrier on which one to three POCIS can be mounted; (**d**) 26 schematic diagram of extraction of analyte from POCIS device.

.
